# Emotionally intelligent school leadership predicts educator well-being before and during a crisis

**DOI:** 10.3389/fpsyg.2023.1159382

**Published:** 2024-02-15

**Authors:** James L. Floman, Annette Ponnock, Jahnvi Jain, Marc A. Brackett

**Affiliations:** Yale Center for Emotional Intelligence, Yale School of Medicine, Yale University, New Haven, CT, United States

**Keywords:** school leader emotional intelligence, leader emotion regulation, leader emotional support, occupational well-being, educator well-being, COVID-19 pandemic, emotion contagion, conservation of resources theory

## Abstract

We examined the role of educator perceptions of school leader emotion regulation (ER) and emotional support (ES) in educator well-being during a typical year and during the COVID-19 pandemic. Based on emotion contagion theory, leaders’ (in)ability to regulate their own emotions may trigger ripple effects of positive or negative emotions throughout their organizations, impacting staff well-being. Additionally, based on conservation of resources theory, when experiencing psychologically taxing events, skillful emotional support provided by leaders may help to replenish staff’s depleted psychological resources, promoting staff well-being. In two national studies, a cross-sectional (*N*_Study 1_ = 4,847) and a two-wave study (*N*_Study 2_ = 2,749), we tested the association between United States preK-12 educator perceptions of school leaders’ ER and ES with educator well-being before and during the COVID-19 pandemic, employing structural equation modeling and multilevel modeling. In Studies 1 and 2, educator reports of their leaders’ ER and ES skills predicted greater educator well-being, including higher positive affect and job satisfaction and lower emotional exhaustion and turnover intentions. In moderation analyses, perceived leader ER predicted well-being about equally among educators facing severe versus mild health impacts from COVID-19. In contrast, perceived leader ES was more strongly associated with educator well-being for some outcomes in those severely versus mildly impacted by COVID-19 illness and death. Leader ER played a role in the well-being of everyone, whereas leader ES was more predictive of well-being for those severely impacted by a crisis. Regarding implications for policy and practice, efforts to promote well-being among educators may be enhanced when combined with efforts to develop school leaders’ ER and ES skills, especially in times of crisis. Accordingly, school districts should consider the value of investing in systematic, evidence-based emotion skills training for their leaders.

## Introduction

The COVID-19 pandemic upended school systems across the globe. In 2020, most schools in the United States converted to part-time or full-time remote instruction with little warning and training, and the most vulnerable students lacked access to critical services and technologies—precipitating short-term and possibly long-term adverse impacts on child health and development (Hamilton et al., 2020; Phelps and Sperry, 2020; Bacher-Hicks et al., 2021; Kaufman et al., 2021). In the years following, staff and students are still engaging in the complex process of settling back into school in person, and reestablishing school norms after being socially distanced for months (Kates et al., 2021; Williams, 2021). Meanwhile, the politicization of education is rising in the U.S., increasing public pressure on leaders and educators, which disrupts school climates and educator well-being (Woo et al., 2022). As a result of these and other factors, educator stress and burnout have escalated (Hamilton et al., 2020; Ferren, 2021; Kim et al., 2022) with stress becoming the leading reason educators report leaving the profession (Diliberti et al., 2021; Steiner and Woo, 2021). Additionally, 72% of school leaders report burnout associated with job stress as a moderate to major concern (Kaufman et al., 2021; see also Brackett et al., 2020; Rubin, 2020). These trends are not only present in schools. About 33% of U.S. adults reported symptoms of anxiety or depression in 2021, and they continue to do so at the end of 2023, compared to about 11% in 2019 before the pandemic [Centers for Disease Control and Prevention (CDC), 2023]. The pandemic exacerbated a national mental health crisis inside and outside of schools (Santoro and Price, 2021).

During times of crisis, leaders play an essential role. They may serve as instruments of support and stability, or they may elevate risk and uncertainty with potential lasting impacts on social cohesion and psychological well-being (Van Vugt et al., 2008; Hannah et al., 2009; Guilaran et al., 2018). In the workplace, emotionally unstable and unsupportive leaders have staff who miss more days of work, are less engaged and satisfied with their jobs, are more likely to quit, and who report more physical and mental health problems (Harris et al., 2009; Kelloway and Barling, 2010; Schyns and Schilling, 2013; Harms et al., 2017; Montano et al., 2017; Nielsen et al., 2017). In the present research, we focused on the extent to which this phenomenon exists in U.S. preK-12 schools. We investigated the role of educator perceptions of their school leaders’ emotion skills in educator well-being, specifically, whether perceived leader emotion regulation (ER) and emotional support (ES) predicted educator well-being before and during the COVID-19 pandemic, a crisis that created an emotionally heightened context in schools (Urick et al., 2021).

With U.S. school leaders and educators facing an array of rising professional (e.g., student learning loss, high turnover rates) and personal stressors (e.g., financial insecurity, parental challenges) brought on by the pandemic (Kaufman et al., 2021; Steiner and Woo, 2021), school leader ER and ES may be significant factors in educator well-being. However, multilevel research investigating the associations between perceptions of school leader ER and ES with educator well-being is limited (Liebowitz and Porter, 2019; Mahfouz et al., 2019; Gómez-Leal et al., 2021). In particular, the role of leader emotion skills in educator well-being during extreme times is currently unclear. It is unknown whether school leader ER and ES matter more during a crisis, and among educators who are most impacted by a crisis. Therefore, we conducted two large-scale national studies, a cross-sectional study run during typical times (*N*_Study1_ = 4,847) and a two-wave study run during the COVID-19 pandemic (*N*_Study2_ = 2,749). We tested whether U.S. educator perceptions of school leaders’ ER and ES predicted educator well-being before and during the pandemic, and whether school leader ER and ES were more predictive of educator well-being among educators more severely impacted by COVID-19 illness and death.

### Defining and measuring occupational well-being in educators

Occupational well-being, including educator well-being specifically, is a broad, multidimensional construct that often includes positively and negatively valenced subjective experiences pertaining to workers’ emotional health, motivation, and engagement (Danna and Griffin, 1999; Day and Qing, 2009; Page and Vella-Brodrick, 2009). As such, educator well-being is typically measured via first-person reports of the following indicators: job satisfaction, perceived stress, positive and negative emotions, burnout, motivation and engagement, and turnover intentions (Fried et al., 2015; McIntyre et al., 2017; Zarate et al., 2019). Given the multidimensionality of occupational well-being, workplace and educator well-being studies often assess the construct with multiple instruments to increase content validity. Based on this literature, we define and measure educator well-being in this research as a single multidimensional construct with facets related to both occupational flourishing (e.g., personal accomplishment, positive affect, and job satisfaction) and occupational languishing (e.g., emotional exhaustion, negative affect, and turnover intentions).

### Leader emotion regulation and educator well-being: theory and evidence

**Figure 1:**
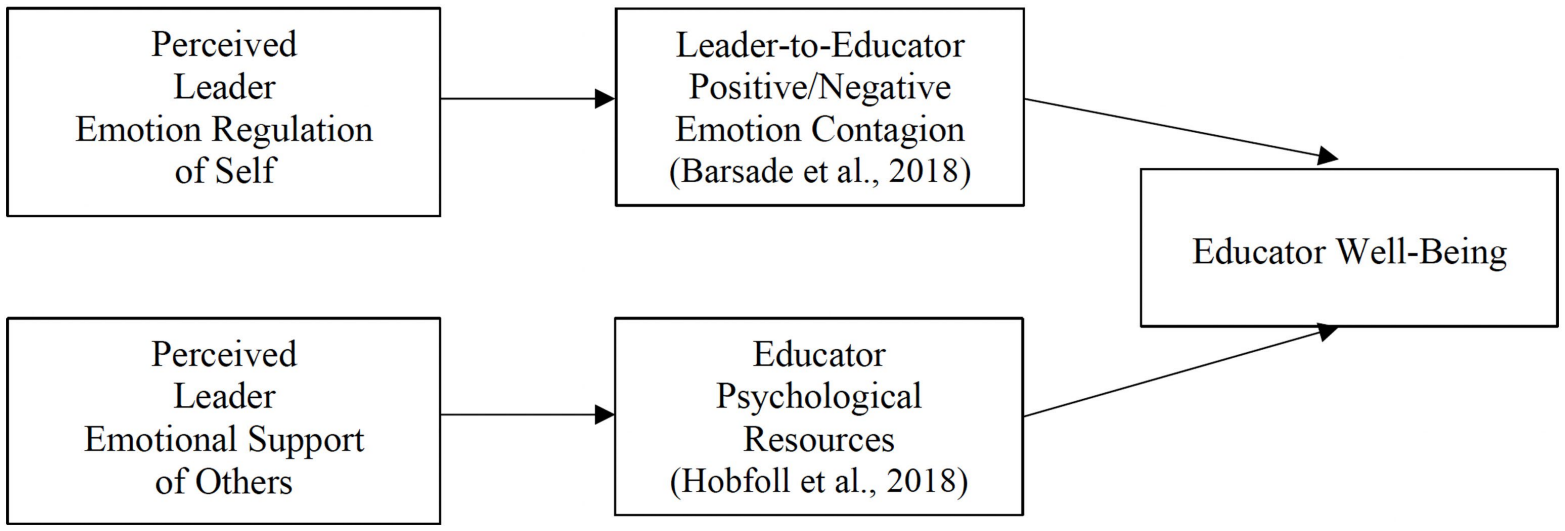
Theoretical model: effects of perceived school leader emotion regulation and emotional support on educator well-being.

One mechanism linking school leader behavior and educator well-being may be leaders’ ability to regulate their own emotions (see Figure 1; George, 2000; Humphrey, 2002; Gooty et al., 2010; Thiel et al., 2015; Liebowitz and Porter, 2019). Emotion regulation is the ability to influence what emotions people experience, when they have emotions, and how they experience and express emotions (Gross, 1999; Mayer et al., 2016). Given the complex organizational and psychological demands of running a school (e.g., managing curriculum and instruction, hiring and staffing, school budgets, and fostering an inspiring school climate), school leaders carry an emotional load that if not regulated puts them at risk of occupational stress, low job satisfaction, and burnout (Darmody and Smyth, 2016; Collie et al., 2020). According to emotion contagion theory, when leaders have difficulty managing their emotions, those emotions can “spread” across organizations (Johnson, 2008; Barsade et al., 2018). That is, when individuals repeatedly interact with one another in shared environments they can both implicitly and explicitly influence each other’s emotional states (Barsade, 2002; Elfenbein, 2014; Chen et al., 2021), as emotional expression and mimicry evolved to rapidly communicate information to the self and others key for survival (Darwin, 1872/1998; Keltner and Haidt, 1999).

Notably, people may be especially sensitive to emotional cues expressed by those with greater power and influence (Sy et al., 2005; Johnson, 2008), and so the emotional state of a school leader may trigger similar emotions in their educators (Bower et al., 2018). As emotions are a central facet of well-being itself (Diener, 2009), and as school leaders may transmit emotions to others who absorb those emotions, leader-driven emotion contagion may play a role in educator well-being. For example, a principal who is visibly anxious about the effects of a pandemic on school health and safety, and cannot effectively down-regulate their feelings, may trigger anxiety in their staff too. Supporting this notion, one study found that principals’ stress levels predicted educators’ stress levels as mediated by educators’ perceptions of their work demands (Westman and Etzion, 1999). Leaders who can manage their own emotions may be less likely to trigger negative emotion contagion in educators, even during a crisis, whereas leaders with ER difficulties may unknowingly spread stress to educators, impacting their well-being.

In line with this reasoning, the staff of transformational and charismatic leaders enjoy greater well-being, possibly due to leaders spreading more positive emotions as well as managing the contagion of negative emotions (Griffith et al., 2015; Harms et al., 2017; Montano et al., 2017). School leaders skilled at *regulating their own emotions* may, for example, up-regulate more inspiration, hope, and pride—and better down-regulate anxiety, anger, and sadness. As such, leaders can shape their school climates to become more imbued with positive versus negative emotions that promote educator well-being in different ways (e.g., reduced emotional exhaustion, increased positive affect and job satisfaction; Mahfouz et al., 2019). This is significant because positive emotions are themselves psychological resources that can promote creative problem solving, cooperation, job performance, and well-being (Lyubomirsky et al., 2005; Zelenski et al., 2008; Diener et al., 2020; Brown and Fredrickson, 2021). Bearing in mind that emotionally skilled leaders know the functional value of all emotions (George, 2000), including negative emotions, they also understand that fostering positive emotions and reducing chronic negative emotions supports occupational well-being and success (Lyubomirsky et al., 2005).

Both inside and outside of educational settings, the evidence linking leader ER skills specifically to staff well-being is limited. One meta-analysis found that organizational leader emotion skills were correlated with a key dimension of occupational well-being: job satisfaction (Miao et al., 2016). However, the studies that reported results for leader ER skills specifically found no effects for ER (only effects averaging across emotion skills). In a large cross-industry study, worker perceptions of their supervisors’ emotion skills were associated with more positive and fewer negative emotions in staff (Ivcevic et al., 2020). Similarly, another study found a positive association between leader emotion skills and staff job satisfaction (Wong and Law, 2002). Yet, in both studies, the results were not reported for ER skills specifically. One novel study did test and report a positive association between staff perceptions of leader ER skills and staff job satisfaction (Zampetakis and Kafetsios, 2010). That said, this study and the other studies mentioned utilized cross-sectional designs, making the direction of association unknown. More satisfied staff may elicit more positive emotions from their leaders (rather than leaders who up-regulate positive emotions promoting job satisfaction in staff), and/or satisfied staff may view their leaders in a more favorable light (a “halo effect”; Forgas and Laham, 2017).

In the school context, four cross-sectional studies report an association between school leaders’ emotion skills and educator job satisfaction. Studies conducted in Indonesia (Waruwu, 2015), Hong Kong (Wong et al., 2010), and Greece (Taliadorou and Pashiardis, 2015) found that school leaders’ emotion skills (self-reported or educator-rated) were positively related to educator job satisfaction. In these studies, however, the relationship between leader ER specifically and educator well-being was not described, so that link remains unclear. Furthermore, in another study conducted in Greece, counter to the studies above, school leaders’ self-reported ER was negatively associated with educator well-being across indicators, including job satisfaction (Kafetsios et al., 2011). The authors suggest that leaders who are “too much in control” of their emotions may seem inauthentic and so may fail to sustain warm, trusting bonds key for educator well-being. Given the limited and conflicting findings, there is a need for research to test the nature and direction of the link between school leader ER and educator well-being. Also, the role of school leader ER in educator well-being during crisis is unknown. To our knowledge, all studies to date test these relationships only during typical times. Educators under extreme stress and most impacted by a crisis might benefit more from leaders skilled in ER, as such leaders may serve as a source of positive emotions and spread fewer negative emotions during challenging times (Urick et al., 2021). We aim to address these gaps to advance knowledge in the field.

### Leader emotional support and educator well-being: theory and evidence

Another mechanism linking school leader behavior and educator well-being may be leaders’ ability to provide emotional support (see Figure 1; George, 2000; Kleine et al., 2019; Liebowitz and Porter, 2019). ES consists of being, “available to listen, to care, to sympathize, to provide reassurance, and to make others feel valued and cared for” (Helgeson, 2003; p. 25), especially in difficult times. Conservation of resources theory holds that the perceived availability of material (e.g., food, medicine) and psychological (e.g., knowledge, skills) resources influences well-being (Hobfoll, 1989; Hobfoll et al., 2018). People focus on minimizing losses and maximizing gains of their resources, and threats to this goal typically trigger stress (e.g., Lesener et al., 2019; Wanberg et al., 2020). The theory suggests that the brain budgets its resources beyond the self to include a network of trusted others, and furthermore, that a major psychological resource is emotional support from trusted others. One of those trusted others might be one’s leader at work (Halbesleben et al., 2014). Moreover, the theory also asserts that in the context of heightened resource loss or threats of loss (e.g., a pandemic), the psychological significance of resource gains increases. Therefore, by collectively shouldering burdens and sharing their psychological resources, school leaders skilled at *supporting others in managing their own emotions* may help to preserve or replenish educators’ psychological resources and offset threats or losses, particularly during a crisis when resources may be more scarce. As a result, educators working for emotionally supportive leaders may experience greater well-being and less ill-being and the salutary effects of leader ES may be enhanced for those who are more impacted by a crisis.

Across industries, perceived supervisor support is consistently and highly associated with job satisfaction and well-being at work (Viswesvaran et al., 1999; Halbesleben, 2006; Kleine et al., 2019). There are two major forms of support leaders provide: emotional support (i.e., listening to emotional experiences, and offering encouragement, recognition, and caring), and instrumental support (i.e., sharing information, opportunities, and resources; Morelli et al., 2015; Jolly et al., 2021). Notably, school leader emotional support predicts educator well-being more than support from colleagues or other sources (Russell et al., 1987; Hughes et al., 2015), and educator’s rate emotional support as the most important form of support (Littrell et al., 1994). This may be the case because of the dynamic, multilayered emotional demands educators face, and school leaders’ ability to modify and reframe educator demands (James et al., 2019; Berkovich and Eyal, 2020). Importantly, educators report receiving emotional support less often than their leaders report offering it, which may undermine educator well-being (Hughes et al., 2015). If addressed, however, this presents an opportunity to promote the well-being of educators.

In prior studies, perceived school leader ES is associated with greater educator well-being and less educator ill-being, including: educators’ ability to regulate their own emotions (Berkovich and Eyal, 2020), burnout (Russell et al., 1987), school commitment, job satisfaction, and physical health (Littrell et al., 1994), stress, self-efficacy, and morale (Lambersky, 2016), psychological needs satisfaction (Ford et al., 2019), turnover intentions and attrition (Podolsky et al., 2016), and quality of life (Yuh and Choi, 2017; for a review, see Liebowitz and Porter, 2019; *cf.* Burke and Greenglass, 1995; Burke et al., 1996). Although these findings are notable, the role of school leader ES during a crisis remains untested, and based on conservation of resources theory, it is under such conditions that leader ES may matter the most for educator well-being (Hobfoll et al., 2018). Further, understanding for whom school leader ES is most helpful has not been well-delineated, and examining who has been most impacted by a crisis will afford an opportunity to examine this question. From the view of conservation of resources theory, those facing the greatest losses or threats are the most likely to benefit from psychological support (Hobfoll et al., 2018). Without subgroup analyses, it is unclear whether perceived leader ES predicts educator well-being for all, or mostly for those facing heightened challenges. Also, prior studies have primarily examined the relationship between school leader ES and educator well-being without linking educator perceptions to their specific leaders (i.e., without using multilevel modeling). We aim to address these limitations.

### The present research

Across two national studies, we tested the hypotheses that U.S. educators who perceived their school leaders as higher on ER (H1) and ES (H2) would report greater well-being. Further, we hypothesized that educator perceptions of their leaders’ ER (H3) and ES (H4) would more strongly predict educator well-being in those severely versus mildly impacted by COVID-19 illness and death. Study 1 employed a large, national sample of U.S. preK-12 educators (*N* = 4,847) with a school-nested subsample, and was conducted before the COVID-19 pandemic in the Spring of 2017. Study 2 extended Study 1 with a two-wave design—to clarify the direction of association between perceived leader ER and ES with educator well-being—using a large, racially diverse sample of U.S. preK-12 educators (*N* = 2,749). In Study 2, we also examined whether perceived leader ER and ES predicted educator well-being during a crisis, and more strongly among those severely impacted by a crisis. Study 2 was conducted in the Fall of 2020 during a national surge in U.S. COVID-19 cases (Johns Hopkins Coronavirus Resource Center, 2021) when schools were heavily disrupted by the pandemic.

## Study 1

### Methods

#### Participants and procedures

The total study sample was *N* = 4,847. School leaders were removed from the sample (*n* = 239), as first-person reports of emotion regulation and emotional support are substantively different from, and may face more threats to validity than, second-person reports (Elfenbein et al., 2015). We report demographics of the remaining analytic sample in Table 1 (*n* = 4,608). Educators were from 48 of 50 U.S. states and one territory (i.e., the District of Columbia). The sample was composed largely of White^1^ (82.6%), female (81.5%), full-time (88.6%) educators and teachers (92.6%)^2^ with a mean age of 43.5 years (*SD* = 11.8) who worked in public schools (66.1%). Average years of experience in education was 15.4 years (*SD* = 10.2) and average years at their current school was 9.2 years (*SD* = 8.1) with a modal annual income of $50,000–$59,999, and a modal level of education reported as a master’s degree (70.5%).^3^

**Table 1:** Study 1: participant demographic characteristics.

Demographic characteristic	% or mean *(SD)*
Age	43.5 (11.8)
Gender	
Female	81.5
Male	17.9
Non-binary identity	0.5
Race/Ethnicity^a^	
White/European American	82.6
Asian/Asian American	7.6
Latinx/Hispanic	6.4
Black/African American	4.8
Other identity	1.8
Native Hawaiian/Pacific Islander	1.4
Native American /Alaskan Native	1.0
Middle Eastern	1.0
State or Territory	
Alabama	0.1
Alaska	0.1
Arizona	0.8
Arkansas	0.2
California	7.1
Colorado	4.2
Connecticut	15.7
Delaware	0.3
District of Columbia	0.3
Florida	2.3
Georgia	0.7
Hawaii	7.8
Idaho	0.2
Illinois	20.1
Indiana	0.3
Iowa	0.2
Kansas	0.2
Kentucky	0.3
Louisiana	0.8
Maine	0.3
Maryland	1.5
Massachusetts	2.1
Michigan	1.3
Minnesota	1.0
Mississippi	0.2
Missouri	0.3
Montana	0.1
Nebraska	1.2
Nevada	0.3
New Hampshire	0.2
New Jersey	2.0
New Mexico	0.2
New York	7.9
North Carolina	0.6
North Dakota	0
Ohio	1.0
Oklahoma	0.9
Oregon	0.3
Pennsylvania	1.3
Rhode Island	0.5
South Carolina	1.2
South Dakota	0.1
Tennessee	1.0
Texas	2.8
Utah	0.5
Vermont	0.3
Virginia	1.0
Washington	5.2
West Virginia	0.1
Wisconsin	0.6
Wyoming	0
Prefer not to say	2.0
Years working in education	15.4 *(10.2)*
Years working in current school	9.2 *(8.1)*
Percent of time employed (FTE)	
Less than 0.25	3.2
0.25–0.49	2.1
0.50–0.74	3.3
0.75–0.99	2.7
1.0	88.6
Extra work hours per week^b^	11.2 *(8.2)*
School type	
Public School	66.1
Independent/Charter School	21.4
Private School	11.5
Religious School	1.0
Average class size	
1–9 students	4.7
10–19 students	28.0
20–29 students	57.7
30–39 students	7.9
40–49 students	0.8
50–59 students	0.1
60 or more students	0.8
Annual income (USD)	
Less than $20,000	3.0
$20,000–$29,999	3.2
$30,000–$39,999	6.9
$40,000–$49,999	13.4
$50,000–$59,999	18.6
$60,000–$69,999	14.2
$70,000–$79,999	11.9
$80,000–$89,999	9.6
$90,000–$99,999	6.2
$100,000–$124,999	9.1
$125,000–$149,999	3.0
$150,000 or more	0.8
School job role	
Educator/Teacher	92.6
School Counselor	4.1
Social Worker	1.5
Psychologist	1.4
Nurse	0.4
Grade level	
Pre-K	7.2
Elementary School	35.5
Middle School	21.2
High School	36.0
Educational attainment	
High School	0.3
Some College (no degree)	1.1
Two-year College	0.8
Bachelor’s Degree	23.2
Master’s Degree	70.5
Doctoral Degree	4.1

*n* = 4,608. This table contains the demographics information for both Studies 1a and 1b. ^a^Percentages do not add up to 100% because respondents could select “all that apply.” ^b^For extra work hours, we asked, “How many hours per week do you currently spend outside of your official work hours doing schoolwork? Please enter only whole numbers from 0 to 50 in the box below”.

An invitation to participate in a study on educator well-being was disseminated online and in person via researcher contacts with school district leaders, non-profits, and national education organizations throughout the U.S. Sharing the study link with educator colleagues was encouraged as well (i.e., snowball sampling). As the study link was distributed widely and shared among informal social networks, we were unable to track all participant recruitment sources. We were able to identify a subset of individuals who participated with their schools, and thus for analysis we separated the sample into a non-school-sourced subsample (Study 1a; *n* = 1793), and a school-sourced subsample (Study 1b; *n* = 2,233, *k* = 88 schools; *M*_educators per school_ = 25.38; *SD*_educators per school_ = 37.10; see Analytic Plan below).^4^ To enhance our ability to study educator well-being at a national level, our sample size was determined by the maximum number of educators interested in participating in the study rather than from conducting a power analysis. Participants took about 20–25 minutes to complete the study measures online via Qualtrics during the Spring of 2017. This study was approved by our university IRB committee.

#### Measures

As this study was part of a larger national study on educator well-being, we employed a series of brief measures. This approach maximized the number of responses and the breadth of construct coverage. The use of short-form measures in large-scale health research is an established practice, including in studies on well-being and happiness where constructs are reliably measured with small sets of items (Cheung and Lucas, 2014; Helliwell et al., 2022). This approach also eased respondent fatigue and reduced the study’s cognitive load, which is important because long surveys may undermine the quality of responses (Egleston et al., 2011; Adams and Umbach, 2012; Borragán et al., 2017). Note that additional measures of educators’ school experiences and health were administered, but they were not included as they are beyond the scope of this study.

##### Perceived school leader emotion regulation and emotional support

We measured school leader ER and ES skills by gathering educators’ global perceptions of their school leaders’ typical ER and ES behavior. Reported perceptions of others’ emotion skills may confer greater predictive validity than self-report or performance measures (Elfenbein et al., 2015; Ivcevic et al., 2020). To parsimoniously obtain educator perceptions of their school leaders’ ER and ES, we created two items to tap ER (i.e., how well leaders manage their own emotions) and two items to tap ES (i.e., how well leaders help others manage their emotions).^5^ The ER items were: “My principal manages their emotions skillfully” and “My principal handles their emotions effectively in stressful situations.” The ES items were: “My principal is emotionally supportive of others” and “My principal is good at helping others feel better when they are upset.”^6^ The response scale was 1 (*completely disagree*) to 6 (*completely agree*). The ER and ES subscales were highly reliable (Spearman-Brown coefficient = 0.95 and = 0.95, respectively).^7^ In the Results, we report a confirmatory factor analysis of our perceived leader ER and ES measure, and the latent correlations between perceived leader ER and ES.

##### Educator well-being measures

We assessed educators’ occupational well-being with indicators of their occupational flourishing (i.e., sense of personal accomplishment, job satisfaction, and positive affect) as well as languishing (i.e., reports of emotional exhaustion, negative affect, and turnover intentions; Danna and Griffin, 1999; Page and Vella-Brodrick, 2009).

###### Emotional exhaustion and personal accomplishment

We used the emotional exhaustion (EE) and personal accomplishment (PAcc) subscales of the Maslach Burnout Inventory for Educators (MBI-ES; Maslach et al., 1996; see also Maslach et al., 2001). The response scale ranges from 1 (*never*) to 7 (*every day*). Example items are: “I feel emotionally drained from my work” (EE) and “I have accomplished many worthwhile things in this job” (PAcc). The EE and PAcc subscales showed high and acceptable reliability: *α* = 0.91 and *α* = 0.79, respectively.

###### Job satisfaction

The three items used to measure job satisfaction were adapted from the Teaching Empowering Leading Learning Survey (TELL Survey; New Teacher Center, 2017). An example item is: “Overall, my school is a good place to work and learn.” The response scale is 1 (*completely disagree*) to 6 (*completely agree*). The scale was highly reliable: *α* = 0.93.

###### Positive affect and negative affect

We assessed positive affect (PA) and negative affect (NA) with the 10-item Positive and Negative Affect Schedule (PANAS; Kercher, 1992; Mackinnon et al., 1999). The PA and NA subscales each contain five items. Example items are: “inspired” and “enthusiastic” (PA) and “nervous” and “distressed” (NA). The response scale is 1 (*never*) to 7 (*always*), indicating how participants felt, “over the past 2 weeks.” Both subscales reached acceptable levels of reliability: *α* = 0.83 (PA) and *α* = 0.85 (NA).

###### Turnover intentions

Turnover intentions were assessed with two items from Moeller et al. (2018): “If an opportunity presented itself, I would pursue another job” and “I have to stay at this job, even though I would rather leave.” The response scale is 1 (*completely disagree*) to 6 (*completely agree*). The measure reliability was acceptable, Spearman-Brown coefficient = 0.72.

###### Educator emotions at school (open-ended)

Participants were asked to: “Think about how you feel each day at school. What are the 3 feelings you experience the most as an educator working in your school? Please write one word in each of the three boxes below.” We combined variants of the same word (e.g., happy, happiness), and removed multi-word phrases, but otherwise left all words unchanged. The top 10 reported emotion words are in Table 2. Open-ended questions may yield information on emotions that close-ended scales fail to capture (Ivcevic et al., 2020) and can reduce common method bias (Podsakoff et al., 2003).

**Table 2:** Study 1: top 10 educator emotions at work (open-ended responses).

“What are the 3 feelings you experience the most as an educator working in your school?”							
1st response		2nd response		3rd response		Overall	
Word	%	Word	%	Word	%	Word	%
Happy	10.46%	Frustrated	11.60%	Frustrated	9.07%	Frustrated	9.63%
Stressed	9.44%	Stressed	6.44%	Tired	5.19%	Happy	6.94%
Excited	9.26%	Happy	5.59%	Stressed	4.93%	Stressed	6.94%
Frustrated	8.22%	Overwhelmed	5.57%	Happy	4.75%	Excited	5.32%
Joyful	7.11%	Excited	4.27%	Overwhelmed	4.73%	Overwhelmed	5.09%
Overwhelmed	4.98%	Anxious	3.53%	Exhausted	3.64%	Joyful	4.23%
Proud	3.39%	Tired	3.24%	Joyful	2.61%	Tired	3.82%
Anxious	3.26%	Joyful	2.96%	Excited	2.41%	Anxious	3.06%
Tired	3.04%	Challenged	2.48%	Anxious	2.39%	Exhausted	2.49%
Inspired	2.28%	Exhausted	2.22%	Challenged	2.10%	Proud	2.40%

*n* = 4,265. Participants were presented with three boxes to describe the top three emotions they experience working at their school. The percentages in the “1st response” to “3rd response” columns refer to responses from each corresponding box. The percentages in the “overall” column refer to participants who reported a specific feeling in 1–3 of the response fields combined. All emotion words were transformed into their adjectival form for presentation purposes, but the emotion word itself was not altered (e.g., “happiness” was changed to “happy”).

##### Covariates

We included covariates in our models that correlate with well-being in educators and other populations (Diener, 2009; Tay et al., 2014; McIntyre et al., 2017). They were: age, gender, race/ethnicity, regular work hours, extra work hours, income, class size, and years working at their school. These covariates also might explain variance in perceptions of school leader ER and ES skills. Educators who feel “overworked and underpaid,” for instance, may rate their leaders’ ER and ES lower because of unfavorable work conditions (e.g., see Burkhauser, 2017). Also, educators who have worked at the same school for longer might know their principal or administrator better, and so may be a more accurate judge of their leader’s ER and ES skills.

### Analytic plan

For our analyses in Study 1, we created two different analytic samples. School-sourced data require an accounting for the non-independence of observations, as the nesting of observations can influence standard errors and bias parameter estimates (Peugh, 2010). The first set of results we report below are from the non-nested sample (Study 1a; *n* = 1793), and the second set of results are from the school-nested sample (Study 1b; *n* = 2,233, *k* = 88 schools).

#### Study 1a and 1b: confirmatory factor analyses

We conducted two sets of confirmatory factor analyses (CFAs). First, to validate the factor structure of the new emotion skills measure, we tested the fit of a single-level (Study 1a) and a multilevel (Study 1b), two-factor model of educator perceptions of school leader ER and ES. Second, despite the multilevel data structure in Study 1b, we conducted single-level CFAs for leader ER and ES and the six multi-item well-being variables^8^ in Study 1b as well, so we could later enter them into a multilevel regression for predictive analyses (see Structural Equation Model and Multilevel Regression Analyses below). We took this approach in Study 1b to reduce the multilevel model (MLM) parameters, so the model would be identified and fit statistics would be produced. As such, variance of the Study 1b factor scores was partitioned at the educator and school level in our predictive analyses rather than in our CFAs.

For our CFAs, we used Mplus 8.5 employing maximum likelihood estimation with robust standard errors (MLR) that addresses multivariate non-normality and data missingness (Muthén and Muthén, 2017). The MLR estimator in Mplus includes full information maximum likelihood (FIML) estimation to handle missing data (Enders and Bandalos, 2001; Shin et al., 2009). For all Study 1a and 1b CFAs, we assessed model fit using the comparative fit index (CFI), the root mean square error of approximation (RMSEA), and standardized root mean squared residual (SRMR; Hu and Bentler, 1999; Hooper et al., 2008). The benchmarks for “adequate fit” were: ≥ 0.90 for CFI, and ≤ 0.08 for RMSEA and SRMR, and for “good fit” were: ≥ 0.95 for CFI, and ≤ 0.05 for RMSEA and SRMR (Hu and Bentler, 1999; Hooper et al., 2008; *cf.* Marsh et al., 2004). Factor loadings above 0.40 were considered acceptable and supportive of the specified factor structure.

#### Study 1a: structural equation modeling

**Figure 2:**
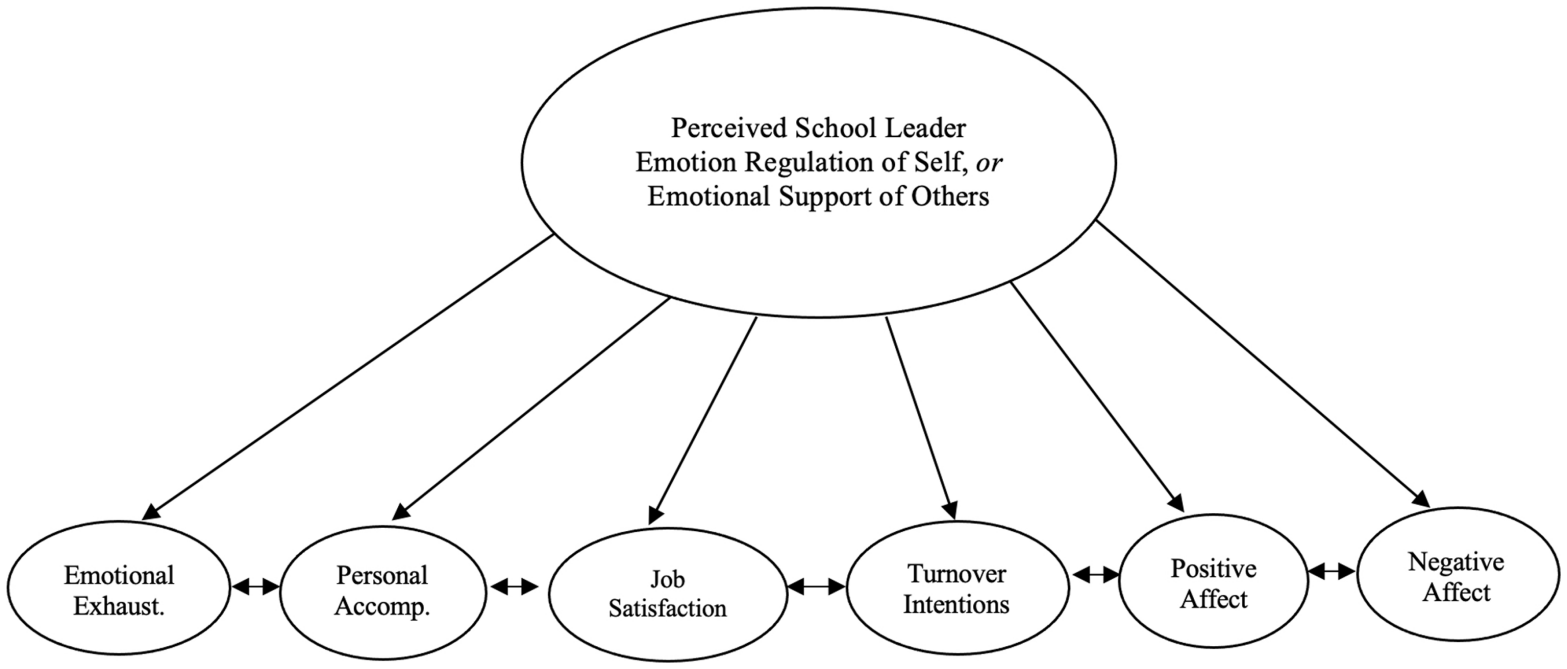
Study 1: perceived school leader emotion regulation or emotional support predicting indicators of educator well-being: simplified statistical model. In Study 1a and 1b, we conducted two main statistical models. In the first, perceived school leader emotion regulation (ER) predicted all educator well-being variables (all outcomes were allowed to covary). In the second, perceived school leader emotional support (ES) predicted all educator well-being variables (all outcomes were allowed to covary). Covariates were also included in these models (see Covariates in the Study 1 Method section). Latent correlations reflecting relationships among key study variables can be found in Table 3 (Study 1a) and Table 4 (Study 1b). To aid readability, covariate paths and item-level paths and error variances for ER, ES, and educator well-being are not depicted here. Circles indicate latent variables. Emotional Exhaust: emotional exhaustion; Personal Accomp: personal accomplishment.

In Study 1a, to test whether perceived school leader ER and ES were associated with educator well-being, we conducted structural equation models (SEMs; see Figure 2). In SEMs, both measurement models (CFAs) and predictive models (path analyses) are typically conducted simultaneously. As such, all multi-item measures were modeled latently using CFA in Study 1a.

That said, in our independent CFAs, the two-factor model of perceived school leader emotion skills showed acceptable fit (see below). Thus, for theoretical and empirical reasons, we modeled leader ER and ES as separate latent variables in our SEMs. However, we did not include leader ER and ES in the same SEM given their high correlation (*r* = 0.86) to reduce multicollinearity which can impact the reliability of parameter estimates (Alin, 2010). We instead conducted two SEMs. In the first model, all latent and observed variables that measured leader ER, educator well-being, and the covariates were entered simultaneously in one SEM. In the second model, all latent and observed variables that measured leader ES, educator well-being, and the covariates were entered simultaneously in another SEM. In our SEMs, the outcomes were allowed to covary, as they all measured educator well-being. Like our CFAs, we used Mplus 8.5 with MLR, and we used the same CFI, RMSEA, and SRMR model fit criteria.

#### Study 1b: multilevel regression analyses

In Study 1b, given the school-nested data structure, we ran two-level multilevel regression models to examine the association between perceived leader ER and ES with educator well-being at L1 (educator level) and L2 (school level). Fewer Type I errors are committed when using MLMs compared to simply adjusting the standard errors to address nesting (Cheah, 2009). An MLM approach also permitted testing whether educators from the same schools perceived their school leaders’ ER and ES similarly (i.e., we report intraclass correlations), and whether perceived leader ER and ES predicted educator well-being outcomes at the individual educator level as well as at the school level. Leaders may influence the collective emotional climate of a school in addition to individuals’ well-being (Mahfouz et al., 2019). Few studies have reported multilevel analyses of leader ER and ES with educator well-being (e.g., Littrell et al., 1994).

That said, MLMs require additional parameters to parse participant-level and cluster-level variance, increasing the demands on the model (Peugh, 2010). As we had 88 clusters—which sets a ceiling on the number of parameters we could include in a single MLM—we took three steps to reduce the model parameters. First, we saved factor scores from the single-level CFAs we conducted and entered those into our multilevel regressions.^9^ Second, we selected a subset of key covariates for our models. For the predictors, we only included one covariate—years working at their school—as educators who are at the same school for longer may have more accurate perceptions of their leaders’ ER and ES. For the outcomes, we included the primary personal demographics only—namely, age, gender, and race/ethnicity—as these are the most widely used covariates in personal and occupational well-being research (Diener, 2009; Tay et al., 2014). Third, we modeled covariates only at the educator level, not at the school level, with one exception. We still regressed perceived leader ER and ES onto years worked at their school at the school level. We did this because schools with educators who have been working there for longer may offer more precise estimates of the relationship between school leader ER and ES skills with collective educator well-being. The Study 1b model is presented in Figure 2.

As with Study 1a, we ran two separate models, one for leader ER and one for leader ES with all well-being outcomes and the aforementioned covariates (see Figure 2). We grandmean-centered the level 1 predictors, which are the same as the level 2 predictors, as this is needed to obtain a meaningful interpretation of intercept and slope parameters in MLMs (Enders and Tofighi, 2007). We used Mplus 8.5 with MLR, CFI, RMSEA, and SRMR as model fit indices, and we specified the “TYPE = Twolevel” command in Mplus for multilevel regressions.

#### Descriptive moderator analyses

Lastly, we examined how the frequency of positive and negative emotion words educators freely generated in describing their feelings about working at school (on open-ended questions) varied by perceived school leader ER and ES skills (at one SD above and one SD below the mean). One member of our research team coded all single emotion words reported into positive (subjectively pleasant) or negative (subjectively unpleasant) emotion categories based on well-studied and supported classifications from the emotion science literature (see Barrett and Russell, 1999; Cowen and Keltner, 2017, 2021). Then, a second researcher reviewed the codes, and 100% agreement was reached through discussion (all disagreements were resolved).

### Results

#### Descriptive statistics and correlations

Descriptive statistics and latent correlations from CFAs between all Study 1 variables are in Table 3 (Study 1a) and Table 4 (Study 1b). Perceived leader ER and ES and a number of demographic factors correlated with indicators of educator well-being in Study 1a and 1b.

**Table 3:** Study 1a: zero-order correlations among latent study variables from CFAs and covariates.

	Perceived leader emotion skills		Educator well-being					
Variable	Emotion regulation	Emotional support	Emotional exhaustion	Personal accomplish	Job satisfaction	Turnover intentions	Positive affect	Negative affect
Covariates								
Age	0.04	0.04	−0.23^***^	0.13^***^	0.13^***^	−0.08^**^	0.17^***^	−0.09^***^
Gender (M/F)	−0.06^*^	−0.04	0.11^***^	0.02	−0.02	0.02	−0.04	0.04
Race/Ethnicity (White/POC)	0.00	−0.02	0.06^*^	0.01	0.01	−0.01	−0.01	−0.05
Regular work hours (Part/Full-Time)	−0.01	−0.02	0.14^**^	0.01	−0.03	0.04	−0.01	0.01
Extra work hours	−0.01	−0.01	−0.01	−0.06^*^	−0.04	−0.03	−0.04	0.03
Income	−0.03	−0.04	−0.03	0.06^*^	0.06^*^	−0.01	0.04	−0.08^**^
Class size	−0.05^*^	−0.05	0.15^**^	−0.05	−0.10^***^	0.08^**^	−0.08^***^	0.09^***^
Years at school	0.01	0.03	−0.14^***^	0.09^***^	0.16^***^	−0.08^**^	0.13^***^	−0.10^***^
Perceived leader emotion skills								
Emotion regulation	—							
Emotional support	0.85^***^	—						
Educator well-being								
Emotional exhaustion	−0.23^***^	−0.27^***^	—					
Personal accomplishment	0.12^***^	0.15^***^	−0.22^***^	—				
Job satisfaction	0.42^***^	0.49^***^	−0.59^***^	0.42^***^	—			
Turnover intentions	−0.30^***^	−0.34^***^	0.50^***^	−0.28^***^	−0.63^***^	—		
Positive affect	0.28^***^	0.31^***^	−0.48^***^	0.47^***^	0.63^***^	−0.48^***^	—	
Negative affect	−0.26^***^	−0.31^***^	0.51^***^	−0.25^***^	−0.53^***^	0.39^***^	−0.43^***^	—

*n* = 1,793. All multi-item measures were modeled latently using CFA. We entered the saved factor scores from the CFAs into bivariate correlations. For extra work hours, responses were positively skewed and so were log-10-transformed. Gender was dichotomized, as the Study 1 subsample reporting non-binary gender identity was small (see Table 1; 0 = male, 1 = female). Race/ethnicity was dichotomized given the small subsample of People of Color [POC; American Psychological Association (APA) (2021)] in Study 1 (see Table 1; 0 = White, 1 = POC). Work hours was dichotomized also as 90.2% of the sample reported working full-time (0 = part-time, 1 = full-time; part-time = 0–0.74 FTE, and full-time = 0.75–1.0 FTE). For binary variables, the last group is the reference. Income was coded from 1 to 12 (1 = less than $20,000 yearly to 12 = $150,000 or more yearly). Class size was coded from 1 to 7 (1 = 1–9 students to 7 = 60 or more students). ^*^p < 0.05. ^⁎⁎^*p* < 0.01. ^⁎⁎⁎^*p* < 0.001.

**Table 4:** Study 1b: zero-order correlations among latent study variables from CFAs and covariates (school-nested sample).

	Perceived leader emotion skills		Educator well-being					
Variable	Emotion regulation	Emotional support	Emotional exhaustion	Personal accomplish	Job satisfaction	Turnover intentions	Positive affect	Negative affect
Covariates								
Age	−0.02	−0.04^*^	−0.19^***^	0.11^***^	0.04^*^	−0.04	0.11^***^	−0.06^*^
Gender (M/F)	0.00	0.00	0.02	0.03	−0.01	0.01	−0.02	−0.01
Race/Ethnicity (White/POC)	0.01	−0.01	0.00	0.03	0.01	−0.06^*^	0.00	−0.06^*^
Regular work hours (Part/Full-Time)	−0.03	−0.04	0.11^***^	0.00	−0.06^*^	0.04^*^	−0.06^*^	0.07^***^
Extra work hours	−0.06^*^	−0.07^**^	0.25^***^	0.04	−0.10^***^	0.03	−0.02	0.14^***^
Income	−0.09^***^	−0.12^***^	0.06^*^	0.02	−0.02	−0.02	−0.06^*^	0.00
Class size	−0.04	−0.07^**^	0.09^***^	−0.03	−0.07^**^	0.03	−0.09^***^	0.05^*^
Years at school	−0.07^***^	−0.09^***^	−0.06^*^	0.07^**^	0.03	−0.01	0.02	−0.03
Perceived leader emotion skills								
Emotion regulation	—							
Emotional support	0.86^***^	—						
Educator well-being								
Emotional exhaustion	−0.27^***^	−0.29^***^	—					
Personal accomplishment	0.13^***^	0.16^***^	−0.23^***^	—				
Job satisfaction	0.39^***^	0.43^***^	−0.63^***^	0.35^***^	—			
Turnover intentions	−0.30^***^	−0.33^***^	0.53^***^	−0.24^***^	−0.67^***^	—		
Positive affect	0.29^***^	0.32^***^	−0.52^***^	0.46^***^	0.64^***^	−0.47^***^	—	
Negative ffect	−0.32^***^	−0.34^***^	0.51^***^	−0.21^***^	−0.52^***^	0.41^***^	−0.41^***^	—

*n* = 2,233. All multi-item measures were modeled latently using CFA. Cluster-adjusted standard errors were used in the CFAs to account for the school-nested data. We entered factor scores from the CFAs into bivariate correlations. For extra work hours, responses were positively skewed and were log-10-transformed. Gender was dichotomized, as the non-binary subsample was small (see Table 1; 0 = male, 1 = female). Race/ethnicity was dichotomized given the small size of POC groups (see Table 1; 0 = White, 1 = POC). Work hours was dichotomized as 90.2% of the sample reported working full-time (0 = part-time, 1 = full-time; part-time = 0–0.74 FTE, full-time = 0.75–1.0 FTE). For binary variables, the last group is the reference group. Income was coded 1–12 (1 = less than $20,000 to 12 = $150,000 or more yearly). Class size was coded from 1 to 7 (1 = 1–9 students to 7 = 60+ students). ^*^p < 0.05. ^⁎⁎^*p* < 0.01. ^⁎⁎⁎^*p* < 0.001.

#### Study 1a: CFA results

In our non-nested sample, the two-factor CFA of perceived leader ER and ES showed good model fit, *X*^2^(1) = 0.34, *p* = 0.56; RMSEA < 0.01; CFI = 1.00; and SRMR < 0.01. The standardized factor loadings were high and ranged from 0.94 to 0.97 for leader ER and 0.93 to 0.95 for leader ES. The latent ER and ES scores correlated strongly (*r* = 0.85, *p <* 0.001). Given the high correlation, we also report a one-factor CFA of perceived leader ER and ES in the Supplementary material. The two-factor model was retained, as it fit the data better, and it aligns with theoretical distinctions that suggest ER and ES may overlap, but are different emotion skills (see Mayer et al., 2016).

#### Study 1a: SEM results

##### Perceived leader ER model

For perceived leader ER, the SEM showed acceptable model fit, *X*^2^(643) = 2724.15, *p* < 0.001; RMSEA = 0.05; CFI = 0.92; SRMR = 0.05, and yielded significant effects (see Table 5). Supporting H1, perceived leader ER predicted less emotional exhaustion and negative affect, fewer turnover intentions, and greater personal accomplishment, job satisfaction, and positive affect (absolute *β*s ranged from 0.12 to 0.42, *p*s < 0.001; see Table 5).

**Table 5:** Study 1a: standardized effects from structural equation modeling (SEM): perceived leader emotion regulation and emotional support predicting educator well-being.

Educator well-being outcomes	Perceived leader emotion regulation			Perceived leader emotional support		
	*β*	*SE*	95% CI	*β*	*SE*	95% CI
Emotional exhaustion	−0.21^***^	0.03	[−0.26, −0.16]	−0.25^***^	0.03	[−0.30, −0.20]
Personal accomplishment	0.12^***^	0.03	[0.06, 0.18]	0.15^***^	0.03	[0.10, 0.21]
Job satisfaction	0.42^***^	0.03	[0.37, 0.47]	0.49^***^	0.02	[0.45, 0.54]
Turnover intentions	−0.35^***^	0.03	[−0.41, −0.29]	−0.41^***^	0.03	[−0.47, −0.35]
Positive affect	0.28^***^	0.03	[0.22, 0.33]	0.31^***^	0.03	[0.26, 0.37]
Negative affect	−0.27^***^	0.03	[−0.33, −0.21]	−0.33^***^	0.03	[−0.39, −0.28]

*n* = 1,793. In Study 1a, separate SEM analyses were conducted for perceived leader emotion regulation (ER) and emotional support (ES) skills to obviate multicollinearity concerns among the predictors. The SEM results reflect perceived leader ER or leader ES predicting all well-being outcomes, and all covariates being regressed onto each predictor as well as all outcomes simultaneously. ^***^p < 0.001.

##### Perceived leader ES model

For perceived leader ES, the SEM also showed acceptable fit, *X*^2^(643) = 2750.60, *p* < 0.001; RMSEA = 0.05; CFI = 0.92; SRMR = 0.05, and indicated significant effects (see Table 5). Supporting H2, perceived leader ES predicted lower emotional exhaustion and negative affect, fewer turnover intentions, and higher personal accomplishment, job satisfaction, and positive affect (absolute *β*s ranged from 0.15 to 0.49, *p*s < 0.001; see Table 5).

#### Study 1b: intraclass correlations

For the school-nested sample, we examined the intraclass correlations (ICCs) to determine how much variance in our main study variables existed at the educator level versus the school level. We found the factor-level ICCs were 0.24 for perceived leader ER and 0.23 for perceived leader ES, indicating about one quarter of the variance in perceived leader ER and ES was at the school level. To some extent, educators at the same school perceived their leaders’ ER and ES similarly. The factor-level ICCs for the educator well-being variables were: 0.11 (emotional exhaustion), 0.03 (personal accomplishment), 0.10 (job satisfaction), 0.09 (positive affect), 0.07 (negative affect), and 0.06 (turnover intentions). These ICCs indicate that educator well-being also may vary by educators’ school. Together, the ICCs suggested we needed to use MLMs in our inferential analyses to parse educator-level and school-level variance (Enders and Tofighi, 2007).

#### Study 1b: CFA results

The two-level, two-factor model of perceived leader ER and ES showed good fit, *X*^2^(2) = 4.03, *p* = 0.13; RMSEA = 0.02; CFI = 1.00; SRMR_within_ < 0.01, SRMR_between_ = 0.01. At the educator level (L1), the standardized factor loadings were high and ranged from 0.94 to 0.94 for leader ER, and 0.93 to 0.94 for leader ES. The latent ER and ES scores showed a large correlation (*r* = 0.85, *p* < 0.001). At the school level (L2), the standardized factor loadings were also high and ranged from 0.98 to 1.00 for leader ER, and 0.99 to 1.00 for leader ES. The latent ER and ES scores showed a large correlation again (*r* = 0.85, *p* < 0.001). Given the high ER–ES correlation, we also report a two-level, one-factor CFA of perceived leader ER and ES in the Supplementary material for completeness. The two-factor model showed better model fit, and is better supported by theory (see Mayer et al., 2016), so it was retained.

#### Study 1b: MLM results

##### Perceived leader ER model

For perceived leader ER, the MLM showed good fit, *X*^2^ (15) = 75.51, *p* < 0.001; RMSEA = 0.05; CFI = 0.99; SRMR_within_ = 0.01; SRMR_between_ = 0.06, and yielded significant effects at L1 and L2 (see Table 6). At L1, supporting H1, perceived leader ER predicted less emotional exhaustion and negative affect, fewer turnover intentions, along with greater personal accomplishment, job satisfaction, and positive affect (absolute *β*s ranged from 0.12 to 0.37, *p*s < 0.001). At L2, supporting H1, perceived leader ER predicted less emotional exhaustion and negative affect, fewer turnover intentions, in addition to greater job satisfaction and positive affect (absolute *β*s ranged from 0.49 to 0.67, *p*s < 0.001). However, the association between perceived leader ER and personal accomplishment was not significant at L2.

**Table 6:** Study 1b: standardized effects from multilevel modeling (MLM): perceived leader emotion regulation and emotional support predicting educator well-being.

Educator well-being outcomes	Perceived leader emotion regulation			Perceived leader emotional support		
	*β*	*SE*	95% CI	*β*	*SE*	95% CI
Educator-level results (L1)						
Emotional exhaustion	−0.23^***^	0.02	[−0.27, −0.20]	−0.26^***^	0.02	[−0.30, −0.23]
Personal accomplishment	0.12^***^	0.03	[0.06, 0.19]	0.16^***^	0.03	[0.10, 0.21]
Job satisfaction	0.37^***^	0.03	[0.32, 0.41]	0.43^***^	0.03	[0.38, 0.48]
Turnover intentions	−0.30^***^	0.02	[−0.33, −0.27]	−0.33^***^	0.02	[−0.36, −0.29]
Positive affect	0.26^***^	0.02	[0.22, 0.30]	0.30^***^	0.02	[0.25, 0.34]
Negative affect	−0.28^***^	0.03	[−0.33, −0.23]	−0.31^***^	0.03	[−0.36, −0.25]
School-level results (L2)						
Emotional exhaustion	−0.49^***^	0.13	[−0.75, −0.24]	−0.51^***^	0.12	[−0.75, −0.27]
Personal accomplishment	0.28	0.24	[−0.20, 0.75]	0.37	0.27	[−0.16, 0.90]
Job satisfaction	0.57^***^	0.11	[0.36, 0.78]	0.57^***^	0.11	[0.36, 0.79]
Turnover intentions	−0.53^***^	0.16	[−0.85, −0.22]	−0.56^***^	0.13	[−0.82, −0.30]
Positive affect	0.53^***^	0.13	[0.29, 0.78]	0.55^***^	0.15	[0.26, 0.85]
Negative affect	−0.67^***^	0.11	[−0.88, −0.45]	−0.70^***^	0.10	[−0.90, −0.51]

Note. n = 2,233, k = 88 schools (M_educators per school_ = 25.38; SD_educators per school_ = 37.10). As with Study 1a, we conducted separate predictive analyses for perceived leader emotion regulation (ER) and emotional support (ES) skills. Given the data in Study 1b were nested within schools, we conducted MLMs. The MLM results reflect perceived leader ER or leader ES predicting all well-being outcomes with years working at their school regressed on each predictor at L1 and L2, and key covariates at L1 (i.e., age, gender, race/ethnicity, and years working at their school) and L2 (i.e., years working at their school) regressed onto all well-being outcomes simultaneously. ^***^p < 0.001.

##### Perceived leader ES model

For perceived leader ES, the MLM also showed good fit, *X*^2^(15) = 146.74, *p* < 0.001; RMSEA = 0.07; CFI = 0.98; SRMR_within_ = 0.01; SRMR_between_ = 0.06, and produced significant effects at L1 and L2 (see Table 6). At L1, supporting H2, perceived leader ES predicted less emotional exhaustion and negative affect, fewer turnover intentions, along with greater personal accomplishment, job satisfaction, and positive affect (absolute *β*s ranged from 0.16 to 0.43, *p*s < 0.001). At L2, supporting H2, perceived leader ES predicted less emotional exhaustion and negative affect, fewer turnover intentions, as well as greater job satisfaction and positive affect (absolute *β*s ranged from 0.51 to 0.70, *p*s < 0.001). However, as with perceived leader ER, perceived leader ES was not significantly associated with personal accomplishment at L2.

#### Study 1a and 1b: descriptive moderation results

**Figure 3:**
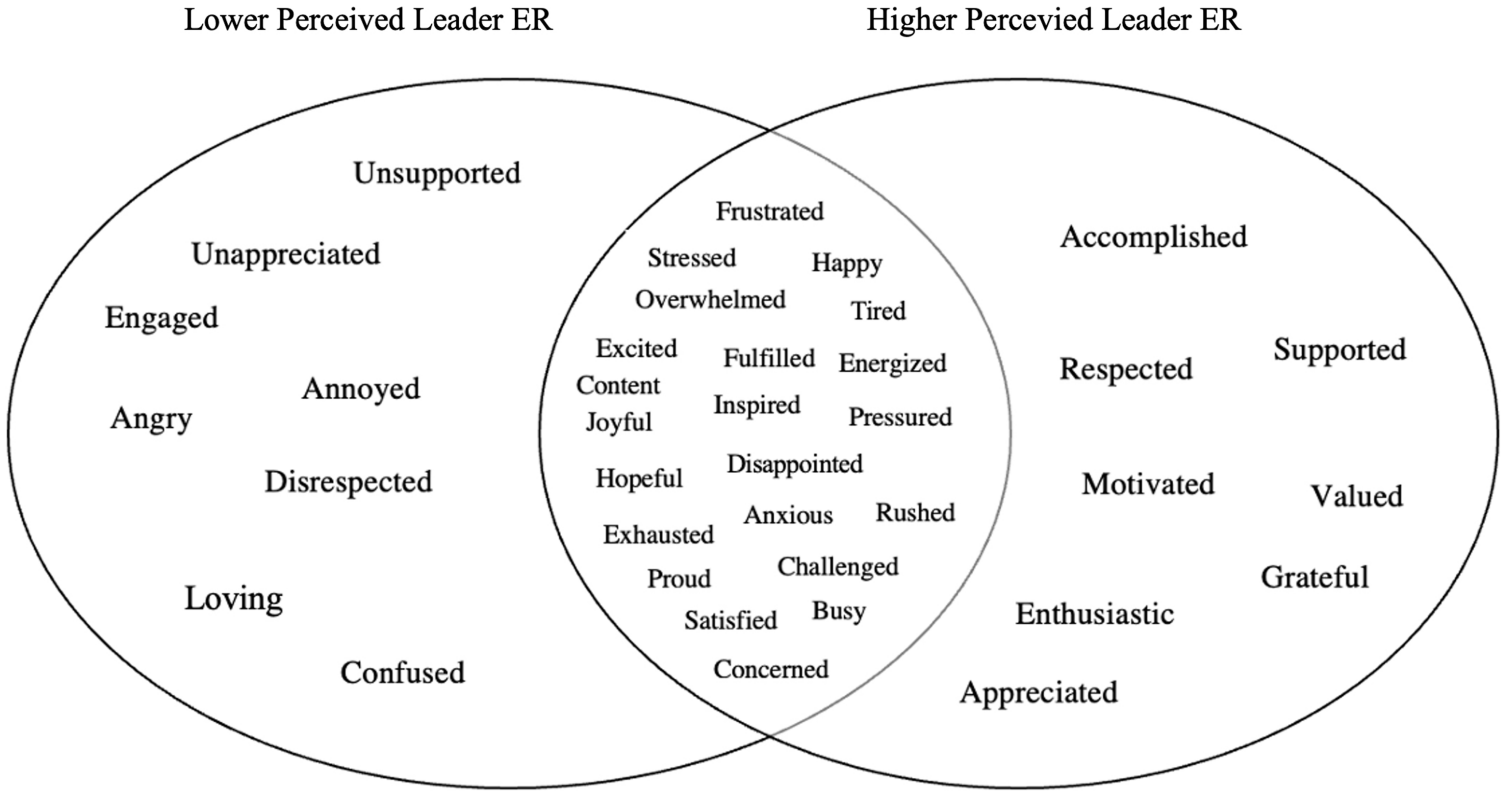
Study 1: top educator emotions (open-ended) as a function of educator perceptions of school leaders’ emotion regulation (ER) skills. *n* = 4,026. The 30 most frequently reported educator emotion words (open-ended) are presented above, as a function of educator perceptions of their school leaders’ ER skills. Lower perceived leader ER skills (the circle on the left) = 1 SD below the mean. Higher perceived leader ER skills (circle on the right) = 1 SD above the mean. The overlap between circles represents shared responses at the mean level of perceived leader ER skills. Responses in the left and the right circles are unique to their respective groups.

**Figure 4:**
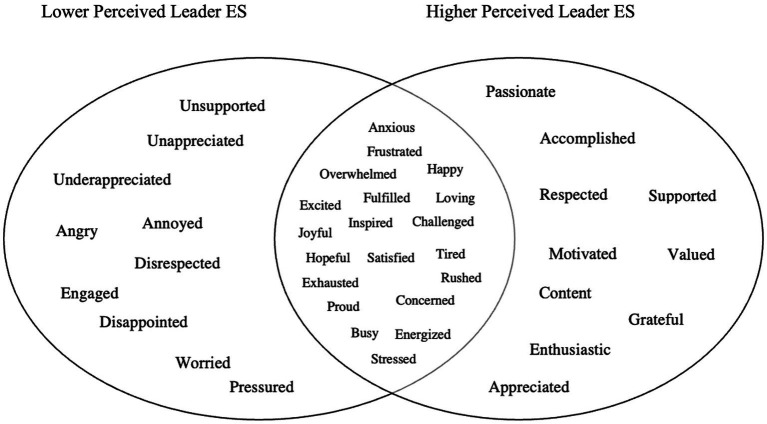
Study 1: top educator emotions (open-ended) as a function of educator perceptions of school leaders’ emotional support (ES) skills. *n* = 4,026. The 30 most frequently reported educator emotion words (open-ended) are presented above, as a function of educator perceptions of their school leaders’ ES skills. Lower perceived leader ES skills (the circle on the left) = 1 SD below the mean. Higher perceived leader ES skills (circle on the right) = 1 SD above the mean. The overlap between circles represents shared responses at the mean level of perceived leader ES skills. Responses in the left and the right circles are unique to their respective groups.

We categorized the top three emotions educators felt at school (collected open-endedly) and explored how they varied by educator perceptions of their leaders’ ER and ES skills. Among all responses, 54.2% were negative emotion and 45.8% were positive emotion words. For educators reporting lower leader ER (one SD below the mean), 66.9% of emotion words were negative and 33.1% were positive. For educators reporting higher leader ER (one SD above the mean), 46.7% of emotion words were negative and 53.3% were positive (see Figure 3). Similarly, for educators reporting lower leader ES, 67.3% of the emotion words were negative and 32.3% were positive. For educators reporting higher leader ES, 45.2% of the emotion words were negative and 54.8% were positive (see Figure 4). These results support both H1 and H2.

### Discussion

In Study 1a, our SEM results indicated that both leader ER and ES predicted less occupational ill-being (i.e., emotional exhaustion, negative affect, and turnover intentions) and greater occupational well-being (i.e., personal accomplishment, job satisfaction, and positive affect) among educators, including personal and school demographics as covariates in the models. In Study 1b, the multilevel regression results for the school-nested sample largely replicated the findings from Study 1a. At the educator level, perceived leader ER and ES predicted higher educator well-being on all six well-being indicators including primary covariates. At the school level, perceived leader ER and ES predicted higher educator well-being on five of the six well-being indicators. Leader ER and ES were not associated with personal accomplishment at the school level, which may be due to the minimal school-level variance in personal accomplishment (ICC = 0.03). The effect sizes were medium in Study 1a and Study 1b at the educator level, while the effects in Study 1b at the school level were medium to large (perhaps because of fewer covariates). We define effect sizes as small (*β* > 0.20), medium (*β* = 0.20–0.49), and large (*β* < 0.50) based on common social science standards (Fey et al., 2023). Educators also generated about 20% more positive emotion words and about 20% fewer negative emotion words to describe their school experience when working for a leader perceived as one SD higher versus lower on ER and ES skills. These effects are akin to, and in some cases larger than, those found in a meta-analysis of average school leader impacts on school outcomes (including on educator well-being; Liebowitz and Porter, 2019), and in recent systematic reviews of leading factors that predict educator well-being (see Hascher and Waber, 2021; Bardach et al., 2022; Wang et al., 2023).

Overall, the SEM, MLM, and open-ended word results support H1 and H2, suggesting that perceived school leader ER and ES specifically are associated with multiple indicators of educator well-being. Furnished with a large national sample, some generalizability of the findings across U.S. educators may be permissible. Yet, Study 1 employed a cross-sectional design, and so the direction of association remains unclear, and educators of color were not well-represented in Study 1, which could limit the representativeness of the findings. Also, Study 1 was conducted during a typical school year, and it did not permit us to test the role of perceived leader ER and ES among educators impacted by a crisis, and whether educators severely versus mildly impacted benefit more from a leader skilled in ER and ES (H3-H4). To address these limitations, we conducted Study 2.

## Study 2

In Study 2, to better understand the direction of association between perceived school leader emotion skills and educator well-being, we used a two-wave design that temporally separated the predictors from the outcomes. We examined the role of educator perceptions of school leader ER and ES in educator well-being during a crisis that severely disrupted the daily functioning of U.S. schools as well as those around the globe (i.e., the COVID-19 pandemic). We hypothesized that perceived school leader ER (H1) and ES (H2) at T1 would predict educator well-being at T2, accounting for covariates. Based on emotion contagion theory, leaders unable to remain calm amidst heightened pressure from the pandemic might be likely to experience and “spread” more negative emotions and fewer positive emotions to their educators (Barsade et al., 2018), who themselves faced increased emotional demands at the time. Based on conservation of resources theory, during a crisis, educator perceptions of their school leaders’ emotional support skills may be uniquely consequential for educator well-being to offset psychological resource losses or threats of loss (Hobfoll et al., 2018). Accordingly, we hypothesized that perceived leader ER (H3) and ES (H4) would predict educator well-being more strongly in educators severely versus mildly impacted by COVID-19 in terms of their exposure to illness and death.

### Methods

#### Participants and procedures

Participant demographics are presented in Table 7 (*n*_T1_ *=* 2,655; *n*_T2_ = 1873).^10^ Participants were from all 50 U.S. states and were mostly White (42.1%), female (54.8%), full-time (70.6%) general or special education teachers^11^ (59.9%) who worked in public schools (72.4%) with a mean age of 37.4 years (*SD =* 8.5). Their average years of experience in education was 9.1 (*SD* = 6.9) with a modal income of $50,000–59,999 a year, and the modal education level was a master’s degree (42.6%). Most educators (59.7%) reported working for schools where learning was hybrid (i.e., working remotely and in-person), while some (32.9%) worked for schools with only remote learning, and a small group (6.9%) worked in schools with only in-person learning.

**Table 7:** Study 2: participant personal and school demographic characteristics.

Demographic characteristic	% or mean (SD)
Age	37.4 (8.5)
Gender	
Female	54.8
Male	45.0
Non-binary identity	0.2
Race/Ethnicity^a^	
White/European American	42.1
Black/African American	37.7
Latinx/Hispanic	23.0
Native American/Alaskan Native	2.1
Asian/Asian American	1.6
Middle Eastern	0.8
Other	0.6
Native Hawaiian/Pacific Islander	0.4
State	
Alabama	0.2
Alaska	0.2
Arizona	3.4
Arkansas	0.5
California	14.7
Colorado	2.0
Connecticut	2.1
Delaware	0.6
Florida	4.8
Georgia	2.5
Hawaii	0.3
Idaho	0.3
Illinois	4.1
Indiana	0.6
Iowa	0.4
Kansas	0.8
Kentucky	0.5
Louisiana	0.7
Maine	0.4
Maryland	1.4
Massachusetts	1.2
Michigan	1.6
Minnesota	1.8
Mississippi	0.3
Missouri	1.3
Montana	0.1
Nebraska	0.3
Nevada	1.4
New Hampshire	0.3
New Jersey	2.5
New Mexico	0.7
New York	8.6
North Carolina	2.2
North Dakota	0.1
Ohio	1.8
Oklahoma	1.1
Oregon	2.7
Pennsylvania	6.7
Rhode Island	0.3
South Carolina	1.1
South Dakota	0.2
Tennessee	1.7
Texas	8.2
Utah	1.2
Vermont	0.4
Virginia	3.0
Washington	5.6
West Virginia	0.3
Wisconsin	2.6
Wyoming	0.1
Years working in a PreK-12 school	9.1 (6.9)
Percentage of time employed	
Less than 0.25	1.0
0.25–0.49	7.8
0.50–0.74	20.6
0.75–0.99	11.9
1.0 (Full-Time)	58.7
Extra work hours	
0 h per day	6.0
1 h per day	14.9
2 h per day	24.5
3 h per day	18.2
4 h per day	14.4
5 h per day	11.6
6 h per day	5.6
7 h per day	2.4
More than 7 h per day	2.4
Annual income (USD)	
Less than $20,000	1.0
$20,000–$29,999	1.8
$30,000–$39,999	4.5
$40,000–$49,999	10.0
$50,000–$59,999	22.4
$60,000–$69,999	19.1
$70,000–$79,999	10.7
$80,000–$89,999	9.3
$90,000–$99,999	6.8
$100,000–$124,999	5.9
$125,000–$149,999	4.0
$150,000 or more	4.5
Job role^a^	
General Education Teacher	52.0
Counselor	12.7
Psychologist	8.7
Instructional Coach	8.7
Special Education/Gifted Education Teacher	7.9
Social Worker	6.3
Other	6.3
After-School Teacher	4.7
Librarian	3.5
Athletic Coach	3.2
Behavior Support Professional	2.7
Administrative Staff	2.7
Paraprofessional	2.2
Technology Specialist	1.7
Nurse	1.1
Grade level^a^	
Pre-K	14.8
Elementary School	57.4
Middle School	38.7
High School	23.2
Education modality	
Remote and in-person student instruction	59.7
Only remote student instruction	32.9
Only in-person student instruction	6.9
Other	0.5

*n* = 2,655. Participants who reported working as a school leader (*n* = 94) were removed from the sample, so they are not included in the statistics reported above. All demographics were reported at T1 (October of 2020). ^a^Percentages do not add up to 100% because respondents could select “all that apply”.

We intentionally oversampled Black (37.7%) and Latinx (23.0%) educators to promote their equitable representation in the study and in research on educator well-being more broadly, which has historically been understudied (e.g., Grooms et al., 2021). We partnered with seven national and regional organizations that work directly with Black and Latinx educators in the U.S. Community partners supported with survey design, messaging, and recruitment efforts. Together, our community partners and the authors disseminated the study via educational organization newsletters, listservs, educational talks and events, and social media for a study on equity in educator well-being. Also, participants were encouraged to contact their colleagues and promote the study as they saw fit (i.e., snowball sampling). To enhance our understanding of educator well-being at a national level, our sample size was determined by the maximum number of educators interested in participating and fiscal resources rather than by a power analysis. The online study took about 20 minutes to complete on Qualtrics in October of 2020 (T1) and December of 2020 (T2) during a surge of COVID-19 cases in the U.S. (Johns Hopkins Coronavirus Resource Center, 2021). This study was approved by our university IRB committee.

#### Measures

Like Study 1, Study 2 was a large-scale national study on educator well-being. As such, to maximize construct coverage and study population-level trends in the U.S. (e.g., Helliwell et al., 2022), while reducing respondent fatigue and the study’s cognitive demands (Egleston et al., 2011; Adams and Umbach, 2012; Borragán et al., 2017), we used mostly short-form measures. Note that other measures of educators’ school experiences and health were completed as part of this research, but they are not reported as they are beyond the scope of this study.

##### Perceived leader emotion regulation and emotional support

Educators reported their perceptions of school leaders’ ER and ES at T1 using the same items as Study 1. The two-factor model of perceived leader ER and ES provided acceptable reliability for ER (Spearman-Brown coefficient = 0.79) and ES (Spearman-Brown coefficient = 0.81). In the Results, we report a CFA of the two-factor model of perceived leader emotion skills to test whether it replicated in Study 2, along with the latent correlation between leader ER and ES.

##### Well-being measures

We assessed educator well-being with largely the same measures used in Study 1 to support commensurability and replicability. We also collected data on COVID-19-related stress and COVID-19 impact, as this study was conducted during a surge in the pandemic. All well-being measure information reported pertains to data collected at T2.

###### Emotional exhaustion

We used the same emotional exhaustion subscale of the MBI-ES (Maslach et al., 1996; see also Maslach et al., 2001) from Study 1, which showed high reliability in this study: *α* = 0.94. For parsimony, we did not include personal accomplishment in Study 2.

###### Job satisfaction

We administered the same job satisfaction measure from the TELL Survey (New Teacher Center, 2017) as Study 1. The measure was moderately reliable: *α* = 0.80.

###### Turnover intentions

We used the same items and response scale as Study 1 to assess turnover intentions (Moeller et al., 2018), but we added one item to increase reliability: “I often think of leaving my current profession.” The scale was moderately reliable: *α* = 0.81.

###### Positive affect and negative affect

Based on emotions open-endedly reported in Study 1, and large-scale open-description emotions research (Ivcevic et al., 2020), we created one five-item measure of positive affect (PA) and one five-item measure of negative affect (NA). We did this as the PANAS has been criticized for capturing primarily high activation emotions (Barrett and Russell, 1999). The five PA items were: “joyful,” “content,” “proud,” “hopeful,” and “inspired.” The five NA items were: “anxious,” “sad,” “angry,” “burned-out,” and “alone.” The response scale was 1 (*none of the time*) to 5 (*all of the time*), indicating how participants felt, “over the past few weeks” (during the COVID-19 pandemic). Reliability was acceptable: *α* = 0.74 (PA) and *α* = 0.73 (NA). In the Results, we report a two-factor CFA of educator affect, and the latent PA-NA correlation.

###### COVID-19 stress

Given the newness of the pandemic when this study was conducted, we created a one-item measure to tap COVID-19-related stress: “Please rate the extent to which the following factors have contributed to your stress over the past month.” One factor was “the COVID-19 pandemic.”^13^ Responses ranged from 1 (*none*) to 5 (*a great deal*).

###### COVID-19 health impact

Participants reported whether they, a loved one, and/or acquaintance contracted COVID-19—and/or a loved one or an acquaintance died from COVID-19. We dichotomized responses: 0 = mild impact (an acquaintance got sick or died, or unaffected; 57.4% of cases) and 1 = severe impact (participant got sick, and/or a loved one got sick or died; 42.6% of cases). Data were collected at T2 during a spike in U.S. COVID-19 cases.

##### Covariates

We measured the following covariates to include in our analyses (see Analytic Plan below) that may account for variance in predictors and outcomes: age, gender, race/ethnicity, regular work hours, extra work hours, income, and distance learning.^14^ These covariates may correlate with occupational and personal well-being in educators and other populations (Diener, 2009; Tay et al., 2014; McIntyre et al., 2017), and they may explain variance in perceptions of leader ER and ES (see Burkhauser, 2017). We also included a measure of distance learning in Study 2, as many students started to attend school from home, at least part of the time, given that schools locked down to reduce the spread of COVID-19. To measure distance learning, we asked: “As of today, how are students learning at your school?” Response options were: “Students are receiving: Only remote/virtual instruction; only in-person instruction; a combination of remote/virtual and in-person instruction; or other (please specify).”

#### Analytic plan

In Study 2, for all analyses, we used Mplus version 8.5 (Muthén and Muthén, 2017) with the MLR estimator (which utilizes FIML) that is largely robust to multivariate non-normality and missingness in the data assuming missing at random (MAR). Data missingness between T1 and T2 was approximately 30% across study variables, which MLR with FIML can handle under conditions of MAR (Shin et al., 2009; Nicholson et al., 2017). Missingness across variables for participants with both T1 and T2 data was low at 2.5%. We conducted three sets of analyses.

First, we conducted a CFA of the two-factor model of perceived school leader emotion skills. We evaluated model fit using RMSEA, CFI, and SRMR, and we also examined the standardized factor loadings. We used the same benchmark values for these criteria that we used in Study 1 to assess model fit and the factor loadings (Hu and Bentler, 1999; Hooper et al., 2008).

**Figure 5:**
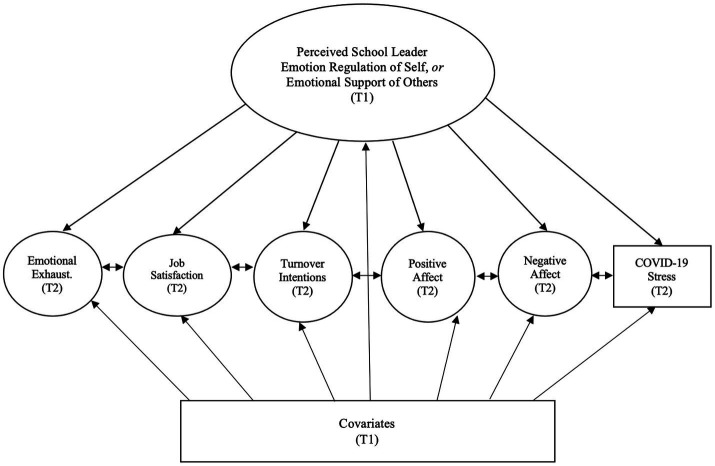
Study 2: perceived school leader emotion regulation and emotional support (T1) predicting indicators of educator well-being (T2): Simplified Structural Equation Model. In Study 2, we conducted two structural equation models (SEMs). In the first, perceived leader emotion regulation (ER) predicted all educator well-being variables (outcomes were allowed to covary), and all covariates (see Covariates in the Method section of Study 2) were regressed onto leader ER along with all well-being outcomes simultaneously. In the second, perceived leader emotional support (ES) predicted all educator well-being variables (outcomes were allowed to covary), and all covariates were regressed onto leader ES along with all well-being outcomes simultaneously. Latent correlations reflecting relationships among key study variables can be found in Table 8. To facilitate readability, item-level paths and error variances are not depicted. Circles indicate latent variables and squares indicate observed variables. Emotional Exhaust., Emotional exhaustion.

Second, we employed SEM to test the extent to which educator perceptions of school leader ER and ES at T1 predicted educator well-being at T2 (H1-H2). All multi-item scales were modeled latently using CFA. Like Study 1, we ran separate SEMs for leader ER and ES, as the strong association between predictors (*r* = 0.86) can cause multicollinearity and reduce the reliability of parameter estimates (Alin, 2010). All educator well-being variables were regressed onto leader ER or ES scores and onto all covariates, while leader ER or ES were regressed onto all covariates simultaneously. The well-being outcomes were permitted to covary (see Figure 5).

Third, we ran moderation analyses in Mplus using an SEM multi-group method (Marsh et al., 2013). This allowed us to test whether educator perceptions of leader ER and ES at T1 predicted educator well-being at T2 more strongly in educators severely versus mildly impacted by COVID-19 (H3-H4).^15^ The model parameters were the same as our main SEMs (including covariates), except we conducted separate models for those with severe and mild COVID-19 health impacts, and then compared the model results to determine moderation using a Wald test.

### Results

#### Descriptive statistics and latent correlations

Descriptive statistics and latent correlations between Study 2 variables are in Table 8. Notably, COVID-19 stress was associated with higher emotional exhaustion (*r* = 0.27, *p* < 0.001), negative affect (*r* = 0.30, *p* < 0.001), and turnover intentions (*r* = 0.06, *p* = 0.01), and lower positive affect (*r* = −0.06, *p* = 0.01). Similarly, experiencing a severe versus mild health impact of COVID-19 was associated with greater COVID-19 stress (*r* = 0.19, *p* < 0.001), emotional exhaustion (*r* = 0.07, *p* = 0.01), and negative affect (*r* = 0.06, *p* = 0.01), and lower positive affect (*r* = −0.05, *p* = 0.03). Interestingly, severe COVID-19 impact also was associated with higher perceived leader ES (*r* = 0.05, *p* = 0.05) and fewer turnover intentions (*r* = −0.06, *p* = 0.02). Overall, these results suggest that the stress and health effects of the pandemic played an adverse role in educator well-being.

**Table 8:** Study 2: zero-order correlations among latent study variables from confirmatory factor analysis and covariates.

	Perceived leader emotion skills (T1)		Educator well-being (T2)					
Variable	Emotion regulation	Emotional support	Emotional exhaustion	COVID-19 stress	Job satisfaction	Turnover intentions	Positive affect	Negative affect
Covariates								
Age	−0.01	−0.01	0.00	0.20^***^	0.04	−0.09^***^	−0.02	−0.07^**^
Gender (M/F)	−0.01	−0.03	−0.01	0.15^***^	0.02	−0.13^***^	−0.08^***^	−0.13^***^
Race/Ethnicity (White/POC)	0.08^***^	0.10^***^	−0.31^***^	−0.12^***^	0.14^***^	−0.12^***^	0.10^***^	−0.15^***^
Regular work hours (Part/Full-Time)	0.03	0.02	−0.09^***^	0.06^**^	0.08^***^	−0.21^***^	0.12^***^	−0.16^***^
Extra work hours	−0.07^**^	−0.08^***^	0.24^***^	−0.04	−0.15^***^	0.23^***^	−0.03	0.19^***^
Income	0.02	0.02	0.16^***^	0.11^**^	0.05^*^	0.13^***^	0.01	0.11^***^
Distance learning (Only Remote/Hybrid & In-Person)	0.01	0.01	0.15^**^	−0.04	−0.05^*^	0.21^***^	0.17^***^	0.09^***^
COVID-19 health impact (Mild/Severe)	0.04	0.05^*^	0.07^**^	0.19^***^	−0.04	−0.06^*^	−0.05^**^	0.06^*^
Perceived leader emotion skills (T1)								
Emotion regulation	—							
Emotional support	0.86^***^	—						
Educator well-being (T2)								
Emotional exhaustion	−0.24^***^	−0.25^***^	—					
COVID-19 stress	0.01	0.00	0.27^***^	—				
Job satisfaction	0.43^***^	0.44^***^	−0.44^***^	−0.04	—			
Turnover intentions	−0.14^***^	−0.15^***^	0.57^***^	0.06^*^	−0.41^***^	—		
Positive affect	0.23^***^	0.24^***^	−0.14^***^	−0.06^**^	0.41^***^	0.01	—	
Negative affect	−0.12^***^	−0.12^***^	0.68^***^	0.30^***^	−0.29^***^	0.48^***^	−0.14^***^	—

*n* = 1,860. All multi-item measures were modeled latently using CFA. Gender was dichotomized, as the subsample reporting non-binary gender identity was small (*n* = 4; 0 = male, 1 = female). Race/ethnicity was measured using a dichotomized variable (0 = White, 1 = POC). Work hours was dichotomized too (0 = part-time, 1 = full-time; part-time = 0–0.74 FTE, full-time = 0.75–1.0 FTE). For extra work hours, we used the same item as Study 1. Income was coded from 1 to 12 for analysis (1 = less than $20,000 yearly to 12 = $150,000 or more yearly). For analytic purposes, we dichotomized distance learning given the distribution of responses: 59.7% (combination remote and in-person instruction); 32.9% (only remote instruction); 6.9% (only in-person instruction); and 0.5% (other). To dichotomize, we combined those working for schools with in-person learning sometimes and all the time (0 = only remote instruction, 1 = combination remote and in-person instruction or only in-person instruction). For COVID-19 health impacts, we dichotomized responses as well: 0 = mild health impact (an acquaintance got sick or died, or unaffected; 57.4% of cases) and 1 = severe health impact (participant got sick, and/or a loved one got sick or died; 42.6% of cases). For binary variables, the last group is the reference group. ^*^p < 0.05. ^⁎⁎^*p* < 0.01. ^⁎⁎⁎^*p* < 0.001.

#### CFA results

##### Perceived leader ER and ES CFA

The two-factor CFA showed good model fit, *X*^2^(1) = 0.14, *p* = 0.71; RMSEA < 0.001; CFI = 1.00; SRMR < 0.001. The standardized factor loadings ranged from 0.80 to 0.81 for perceived leader ER, and 0.82 to 0.84 for perceived leader ES. The ER and ES factor scores correlated highly (*r* = 0.86, *p < *0.001). Given this correlation, we also report a one-factor CFA of perceived leader emotion skills in the Supplementary material. The two-factor model fit relevant theory (see Mayer et al., 2016) and the data better, and thus it was retained.

##### Positive and negative affect CFA

We conducted a two-factor CFA of our new PA and NA measure (see Barrett and Russell, 1999). The CFA for PA and NA showed acceptable model fit, *X*^2^(34) = 211.30, *p* < 0.001; RMSEA = 0.05; CFI = 0.94; SRMR = 0.04. The standardized factor loadings ranged from 0.55 to 0.70 for PA, and 0.53 to 0.62 for NA. PA and NA factor scores showed a small inverse correlation (*r* = −0.11, *p = *0.01). We retained the two-factor model of affect.

##### SEM analyses

The perceived leader ER SEM showed acceptable model fit, *X*^2^ (412) = 2062.69, *p* < 0.001; RMSEA = 0.05; CFI = 0.92; SRMR = 0.05, and indicated significant effects (see Table 9). Supporting H1, leader ER at T1 predicted less educator emotional exhaustion and negative affect, fewer turnover intentions, along with greater job satisfaction and positive affect at T2 (absolute *β*s ranged from 0.11 to 0.48, *p*s < 0.001). Leader ER did not predict COVID-19 stress.

**Table 9:** Study 2: standardized effects from structural equal modeling (SEM): perceived leader emotion regulation and emotional support (T1) predicting educator well-being (T2).

Educator well-being outcomes (T2)	Perceived leader emotion regulation (T1)			Perceived leader emotional support (T1)		
	*β*	*SE*	95% CI	*β*	*SE*	95% CI
Emotional exhaustion	−0.21^***^	0.03	[−0.26, −0.16]	−0.23^***^	0.03	[−0.28, −0.18]
Job satisfaction	0.48^***^	0.03	[0.42, 0.54]	0.50^***^	0.03	[0.44, 0.56]
Turnover intentions	−0.13^***^	0.03	[−0.19, −0.07]	−0.16^***^	0.03	[−0.22, −0.10]
Positive affect	0.27^***^	0.03	[0.21, 0.33]	0.27^***^	0.03	[0.21, 0.33]
Negative affect	−0.11^***^	0.03	[−0.18, −0.05]	−0.13^***^	0.03	[−0.19, −0.06]
COVID-19 stress	0.03	0.03	[−0.03, 0.08]	0.01	0.03	[−0.04, 0.06]

*n* = 1,860. In Study 2, as in Study 1, separate SEMs were conducted for perceived leader ER and leader ES given concerns about multicollinearity among the predictors. The SEM results reflect perceived leader ER or leader ES predicting all well-being outcomes, and all covariates being regressed onto each predictor and all outcomes simultaneously. ^***^p < 0.001.

The perceived leader ES SEM also showed acceptable model fit, *X*^2^(412) = 2051.73, *p* < 0.001; RMSEA = 0.05; CFI = 0.92; SRMR = 0.05, and produced significant effects (see Table 9). Supporting H2, leader ES at T1 predicted lower emotional exhaustion and negative affect, fewer turnover intentions, and higher job satisfaction and positive affect at T2 (absolute *β*s ranged from 0.13 to 0.50, *p*s < 0.001). Perceived leader ES also was unrelated to COVID-19 stress.

##### SEM moderation analyses

We ran a multi-group SEM analysis with COVID-19 impact as a dichotomous moderator. We compared a model where the slopes for the severe COVID-19 impact group were constrained to be equal to the mild COVID-19 impact group with a model where the slopes were free to vary. The chi-squared difference test between the constrained (*X*^2^ = 4414.73, *p* < 0.001; RMSEA = 0.06; CFI = 0.88; SRMR = 0.09) and unconstrained model (*X*^2^ = 3524.45, *p <* 0.001; RMSEA = 0.05; CFI = 0.91; SRMR = 0.06) was significant (*p* < 0.001), indicating an overall moderation effect. We then conducted models freeing each constrained structural path one at a time to test for moderation of the link between leader ER, and separately for leader ES, with each educator well-being outcome. We utilized a Wald chi-square test in Mplus with the “MODEL TEST” command to test for moderation (Muthén and Muthén, 2017). We report significant moderation results below.

###### Moderation of perceived leader ER and positive affect

The test of moderation for perceived leader ER predicting positive affect was significant, *X*^2^(108) = 375.98, *p* < 0.001; RMSEA = 0.05; CFI = 0.91; SRMR = 0.06; Wald test(1) = 8.07, *p* = 0.01. Counter to our hypothesis (H3), leader ER predicted positive affect more strongly in the mild (*β* = 0.35, *p* = 0.00) vs. the severe (*β* = 0.19, *p* = 0.00) COVID-19 impact group. Potential reasons for this are offered below.

###### Moderation of perceived leader ER and turnover intentions

The test of moderation for perceived leader ER predicting turnover intentions was just shy of significance, *X*^2^(58) = 296.68, *p* < 0.001; RMSEA = 0.07; CFI = 0.93; SRMR = 0.07; Wald test(1) = 3.69, *p* = 0.06. Consistent with our hypothesis (H3), leader ER predicted turnover intentions more strongly in the severe (*β* = −0.22, *p* = 0.00) vs. the mild (*β* = −0.09, *p* = 0.04) COVID-19 impact group.

###### Moderation of perceived leader ES and emotional exhaustion

The test of moderation for perceived leader ES predicting emotional exhaustion was significant, *X*^2^(166) = 765.01, *p* < 0.001; RMSEA = 0.06; CFI = 0.94; SRMR = 0.06; Wald test(1) = 6.82, *p* = 0.01. Supporting our hypothesis (H4), leader ES predicted emotional exhaustion more strongly in the severe (*β* = −0.31, *p* = 0.00) vs. the mild (*β* = −0.17, *p* = 0.00) COVID-19 impact group.

###### Moderation of perceived leader ES and negative affect

The test of moderation for perceived leader ES predicting negative affect also was significant, *X*^2^(108) = 473.96, *p* < 0.001; RMSEA = 0.06; CFI = 0.88; SRMR = 0.06; Wald test(1) = 8.06, *p* = 0.01. Consistent with our hypothesis (H4), leader ES predicted negative affect more strongly in the severe (*β* = −0.23, *p* = 0.00) vs. the mild (*β* = −0.04, *p* = 0.40) COVID-19 impact group. Notably, the effect was significant in the severe impact group, but not significant in the mild impact group.

###### Moderation of perceived leader ES and turnover intentions

**Figure 6:**
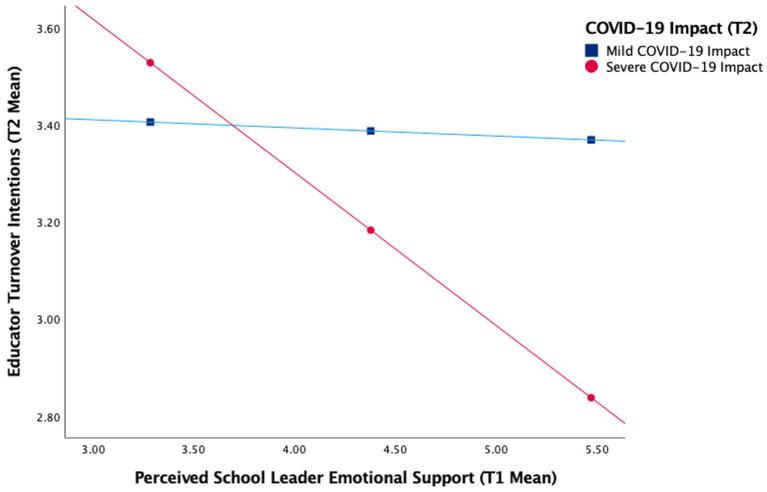
Study 2 moderation analysis: perceived school leader emotional support (T1) predicting educator turnover intentions (T2) as a function of COVID-19 health impact (T2) 3.60 COVID-19 impact (T2) mild COVID-19 impact severe COVID-19 impact educator turnover intentions (T2 Mean). *n* = 1841. The three points along each graph line depict levels of perceived school leader emotional support: 1 SD below the mean, the mean, and 1 SD above the mean. We dichotomized responses to the COVID-19 impact question: 0 = mild COVID-19 impact (an acquaintance got sick or died, and/or unaffected; 57.4% of cases) and 1 = severe COVID-19 impact (participant got sick, and/or a loved one got sick or died; 42.6% of cases). The terms “mild” and “severe” are relative and indicate COVID-19 impacts on illness and death only. A multi-group structural equation model analysis indicated a significant moderation effect for turnover intentions along with other well-being outcomes (see SEM Moderation Analyses in the Study 2 Results section).

The test of moderation for perceived leader ES predicting turnover intentions was significant, *X*^2^(58) = 311.79, *p* < 0.001; RMSEA = 0.07; CFI = 0.93; SRMR = 0.07; Wald test(1) = 12.47, *p* < 0.001. Supporting our hypothesis (H4), leader ES predicted turnover intentions more strongly in the severe (*β* = −0.28, *p* = 0.00) versus the mild (*β* = −0.07, *p* = 0.12) COVID-19 impact group. As with negative affect, this effect was significant in the severe impact group, but not in the mild impact group. We depict the results of this analysis in Figure 6 as an example illustration of the moderation pattern.

### Discussion

In Study 2, we found that 42.6% of the sample had either contracted COVID-19 or a loved one had contracted and/or died from COVID-19. Those dealing with these impacts of the pandemic versus those who were spared, showed increased COVID-19 stress, and poorer well-being across multiple indicators, including their positive and negative affect. With that context in mind, consistent with the findings from Study 1, educator perceptions of their school leaders’ ER at T1 predicted less emotional exhaustion and negative affect, fewer turnover intentions, and greater job satisfaction and positive affect at T2 (supporting H1). Likewise, consistent with the Study 1 results, educator perceptions of their school leaders’ ES at T1 also predicted lower emotional exhaustion and negative affect, fewer turnover intentions, and higher job satisfaction and positive affect at T2 (supporting H2). The significant effects observed in Study 2 were small to medium in size, similar to the effects in Study 1 (Fey et al., 2023). These effects are commensurate with those reported in empirical reviews of school leader impacts on educators and students (Liebowitz and Porter, 2019), and reviews of primary predictors of educator well-being (see Hascher and Waber, 2021; Bardach et al., 2022; Wang et al., 2023). That said, neither perceived leader ER nor ES predicted COVID-19 stress in educators at the height of the COVID-19 pandemic in the U.S. Although unknown, more proximal COVID-19 risk factors may have driven COVID-19 stress, including health vulnerabilities, access to health care resources, and exposures to physical, financial, and emotional threats of the pandemic.

Additionally, the negative associations between perceived leader ER and ES with turnover intentions were stronger in educators severely versus mildly impacted by COVID-19 illness and death (supporting H3 and H4). Similarly, the negative links between leader ES with emotional exhaustion and negative affect were larger in those severely versus mildly impacted by COVID-19 (supporting H4). These results suggest that leader ER and ES skills were more helpful for educators facing the worst of the pandemic, at least for some well-being outcomes. That said, a number of moderation analyses were not significant and it is not clear why some outcomes showed moderation and others did not. We also found, counter to our hypothesis, that perceived leader ER predicted positive affect more strongly in educators mildly versus severely impacted by COVID-19 (counter to H3). Perhaps educators who were able to avoid the worst health effects of the pandemic were better able to appreciate and/or absorb their leader’s positive emotions than those who did not. Taken together, the results provide partial support for both emotion contagion and conservation of resources theories, which suggest that during a crisis, leaders who are more emotionally regulated may mitigate negative emotion contagion and promote positive emotions (Barsade et al., 2018), and leaders who provide emotional support may help to offset threats or losses through the collective sharing of psychological resources (Hobfoll et al., 2018).

## General discussion

What roles do emotion regulation and emotional support play in effective leadership, especially during times of crisis? We conducted two large-scale national studies in the U.S. to address this question, examining the relationships between school leaders’ ER and ES skills with educator well-being before and during the COVID-19 pandemic. In Study 1, educators freely generated about 20% more positive emotion words and 20% fewer negative emotion words to describe their school experience when working for a leader perceived as one standard deviation higher on ER and ES (supporting H1 and H2). We also found that perceptions of leaders’ ability to manage their own emotions were associated with higher educator well-being during typical times (Study 1) and the pandemic (Study 2) (supporting H1). This included less emotional exhaustion and negative affect, fewer turnover intentions, and greater job satisfaction and positive affect. Similarly, perceptions of leaders’ ability to provide effective emotional support predicted greater educator well-being on the same outcomes in Studies 1 and 2 (supporting H2).

Additionally, in Study 2, the effects of perceived leader ER and ES depended (in part) on COVID-19 impact. Perceived leader ER predicted positive affect only in educators mildly impacted by COVID-19 (counter to H3), whereas leader ER predicted fewer turnover intentions more strongly in educators severely impacted by COVID-19 (supporting H3). Also, perceived leader ES predicted lower emotional exhaustion, negative affect, and turnover intentions more strongly in educators severely versus mildly impacted by COVID-19 (supporting H4). As such, perceived leader ES appeared to be uniquely valuable for the educators most affected by the pandemic. Educators who were ill, and/or had a loved one who was ill or died from COVID-19, experienced significantly less emotional exhaustion and negative affect, and were less likely to want to quit their jobs if they saw their leader as more emotionally supportive. These findings are novel, as past studies on school leader ER and ES were not conducted during a crisis.

Why did we observe this pattern of effects? For perceived leader ER, based on emotion contagion theory (Barsade et al., 2018), school leaders who routinely up-regulate positive emotions may intentionally and unintentionally “spread” joy and inspiration to educators. This may then boost educator daily positive affect and well-being (Fredrickson, 2013; Diener et al., 2020). Conversely, school leaders who fail to regulate their negative emotions while meeting a host of demands may create negative emotion contagion that raises anxiety and stress at their school, increasing educator ill-being, especially during times of crisis. Our findings from Studies 1 and 2 support these notions, as educators working for leaders with more developed ER skills showed higher well-being (e.g., job satisfaction) and lower ill-being (e.g., emotional exhaustion) across multiple indicators. That said, COVID-19 health impacts largely did not moderate the role of perceived leader ER in educator well-being. It may be that the effects of leader ER-driven emotion contagion are common and pervasive, and so they persist regardless of an individual’s exposure to additional stressors. The results of our moderations support this idea. Perceived leader ER predicted almost all educator well-being outcomes in both the mild and severe COVID-19 impact groups. Moderation was lacking not because effects were absent, but due to approximately equivalent effects in each of the COVID-19 impact groups.

For perceived leader ES, we can understand our findings with conservation of resources theory (Hobfoll, 1989; Hobfoll et al., 2018). The theory contends that an individual’s material (e.g., food, medicine) and psychological (e.g., knowledge, skills) resources are budgeted by the brain across a dynamic network of trusted others. Resource losses trigger stress, but because resources are perceived as shared across a network, when someone faces resource losses, others in their network can help to make up the difference. People with more power and resources in a network may play an outsized role in providing such support, particularly in times of crisis when resources are scarce. Accordingly, trusted leaders may help to ease the emotional load of staff by sharing their psychological resources, effectively reducing their staff’s sense of psychological resource depletion and expanding their perceived ability to cope, especially during a crisis. This is largely what we found. Both before (Study 1) and during (Study 2) the COVID-19 pandemic, perceived leader ES predicted higher educator well-being and lower educator ill-being. Further, in Study 2, perceived leader ES predicted less emotional exhaustion more strongly in the severe versus the mild COVID-19 impact group. Also, perceived leader ES only predicted lower negative affect and turnover intentions in the severe COVID-19 impact group – the paths were not significant in the mild COVID-19 impact group. These findings suggest that perceived leader ES might matter more for the well-being of educators facing increased psychological demands and losses, which is aligned with the principles of conservation of resources theory. That said, the relationships between leader ES and other educator outcomes were not moderated by the degree of crisis exposure, specifically, the positive indicators of well-being (job satisfaction and positive affect). It may be that leader ES is of greater importance for indicators of ill-being among those facing the worst of a crisis.

Our findings are broadly consistent with prior studies on the role of leader emotion skills in occupational well-being. Past studies suggest that leaders’ emotion skills are positively associated with a key indicator of occupational well-being—job satisfaction—both among educators in schools (Wong et al., 2010; Taliadorou and Pashiardis, 2015; Waruwu, 2015; *cf.* Kafetsios et al., 2011), and among individuals from other contexts (Wong and Law, 2002; Zampetakis and Kafetsios, 2010; Miao et al., 2016; Ivcevic et al., 2020). Only two studies reported the relationship between leaders’ ER skills with occupational well-being. One study found that leader ER was related to higher staff job satisfaction (Zampetakis and Kafetsios, 2010), and the other found that leader ER was related to lower educator job satisfaction (Kafetsios et al., 2011). These conflicting studies used cross-sectional designs and job satisfaction as the primary index of well-being, limiting the conclusions one could draw about leader ER.

Several studies have examined leader ES in occupational and educator well-being (Liebowitz and Porter, 2019) with most studies finding that greater school leader ES is associated with higher educator well-being (Russell et al., 1987; Littrell et al., 1994; Lambersky, 2016; Podolsky et al., 2016; Yuh and Choi, 2017; Ford et al., 2019; Berkovich and Eyal, 2020; *cf.* Burke and Greenglass, 1995; Burke et al., 1996). However, none of the prior studies investigated whether these effects existed during a time of crisis—when ES might matter the most—and whether those more exposed to a crisis may benefit more from a leader skilled in ES. Our findings suggest that leader ES may matter more for those facing increased challenges rather than for everyone, at least during a crisis on indicators of ill-being. Prior effects showing the value of leader ES for staff well-being might be driven (in part) by subgroups facing higher distress. Also, few studies have used multilevel modeling to parse educator-level and school-level variance in testing the relationships between school leader ER and ES with educator well-being. As such, these studies are the first to our knowledge that report the associations between perceived school leader ER and ES with multiple indicators of educator well-being using cross-sectional and two-wave designs, multilevel modeling, and SEM-based moderation. We do so across two large-scale national studies, one of which includes educators from racially diverse backgrounds collected during a global pandemic. Our results offer empirical support for the idea that school leaders may play a role in educator well-being via the emotion skills (ER and ES) they enact in their organizations (Mahfouz et al., 2019).

### Study limitations and future research directions

This research has limitations that should be noted when interpreting our findings. First, in Study 1, the design was cross-sectional, and so the directionality of effects is unclear. In Study 2, we employed a two-wave design to test directionality; however, our data are non-experimental, and so causal inferences cannot be drawn. Future studies using controlled intervention designs that test for training effects on leaders’ emotion skills, and examine their downstream effects on occupational well-being are needed (e.g., Mahfouz, 2018). Second, although perceptions of others’ emotion skills may be less subject to self-enhancement biases than self-reports and are more ecologically valid than some performance measures (Elfenbein et al., 2015), all data were sourced from educators. Future work might use 360-degree informant ratings and performance measures of leader emotion skills, and then link those measures to educator well-being. Also, given all educator well-being outcomes were self-reported, future research might collect second-person ratings of educator well-being-related behaviors and third-person metrics, such as records of absences and attrition rates. Third, to suit the large-scale national research goals of Studies 1 and 2, we utilized a new short-form measure to assess perceived school leader ER and ES. We reported the results of a preliminary validity study of our measure, but this study may not have fully mapped the latent structure underlying the phenomena, and more evidence of construct validity (including convergent and discriminant validity) is needed to evaluate the measure before widespread adoption. A promising future direction would be to expand the perceived school leader ER and ES measures and conduct psychometric studies to build on this work. Lastly, although Studies 1 and 2 recruited large-scale national samples in the U.S., there may be selection effects regarding who participated in these studies and those who did not. Relatedly, the pattern of findings may not generalize to educators working outside of the U.S. As noted, the politicization of education and public pressure on educators in the U.S. might contribute to stressful working conditions and exacerbate burnout, and so future research should explore the links between school leader ER and ES with educator well-being in other countries.

### Research implications for policy and practice

Crises reveal and magnify fractures in ourselves and our institutions. By the end of 2023, U.S. adults reported about a threefold increase in anxiety and depression symptoms compared to reports from 2019 [Centers for Disease Control and Prevention (CDC), 2023]. School leaders and educators face chronic stress triggered by the COVID-19 pandemic, as well as perennial challenges to education systems, and in the U.S., increasing public pressure from efforts to politicize education (Hamilton et al., 2020; Ferren, 2021; Kaufman et al., 2021; Steiner and Woo, 2021; Woo et al., 2022). Our findings suggest that in U.S. schools, developing leaders’ ER and ES skills to care for overloaded faculty and staff may prove useful in reducing emotional exhaustion, negative affect, and turnover intentions, along with promoting personal accomplishment, positive affect, and job satisfaction. Investments in these trainings may mitigate the rising psychological, educational, and financial costs of educator burnout and attrition. Prior studies have found that the ER and ES skills of school leaders and educators can be enhanced via systematic, evidence-based trainings (Klingbeil and Renshaw, 2018; Mahfouz, 2018; Gómez-Leal et al., 2021). Policies that promote and fund such leadership development training should be considered in light of not just our findings, but the growing body of evidence regarding leader impacts on school climate and various metrics of success, including student achievement (e.g., Liebowitz and Porter, 2019). Importantly, leadership programs can be offered to those training to become leaders and to those currently serving in leadership positions. Offering training in both places will help to realize the positive impact potential of educational leaders. As the COVID-19 pandemic in the U.S. and around the world wanes, its effects on school systems and educators may still linger and even compound. Developing leaders’ ER and ES skills shows promise in helping schools to build their capacity to weather present and future storms.

## Data availability statement

The raw data supporting the conclusions of this article will be made available by the authors upon request for research purposes without undue reservation.

## Ethics statement

The studies involving humans were approved by the Yale University Institutional Review Board. The studies were conducted in accordance with the local legislation and institutional requirements. The participants provided their informed consent to participate in this study.

## Author contributions

JF designed and selected measures for Study 1 with MB and Study 2 with AP and MB. JF programmed the measures into Qualtrics for Studies 1 and 2 with support from AP. JF and MB were involved in study recruitment for Study 1. JF, AP, and MB were involved in study recruitment for Study 2. JF primarily conducted all analyses across Studies 1 and 2 with support from AP. JJ coded the open-ended emotions data in Study 1 and helped to prepare tables and figures for Studies 1 and 2. JF composed the majority of the manuscript (all sections) with support and comments from AP and MB. All authors contributed to the article and approved the submitted version.

## Footnotes

^1^Race/ethnicity terminology is based on the American Psychological Association (APA) (2021) Inclusive Language Guidelines. The terms used in the main text may refer to identities with shorter labels for economy, though the terms used in Table 1 (Study 1) and Table 7 (Study 2) do not do so. These terms are: Black/African American, Latinx/Hispanic, and White/European American.

^2^We invited all school faculty and staff to participate, as school leaders may impact the well-being of all school personnel. For convenience, we refer to all school personnel as “educators” throughout the paper.

^3^A higher percentage of United States preK-12 educators hold master’s degrees than the general United States population [U.S Census Bureau, 2020; National Center for Education Statistics (NCES), 2023].

^4^The total Study 1a and Study 1b sample sizes were *n* = 2,102 and *n* = 2,506, respectively. However, *n* = 309 (Study 1a) and *n* = 273 (Study 1b) participants showed missingness on all close-ended variables. As such, the subsamples for Study 1a and Study 1b were *n* = 1793 and *n* = 2,233, respectively. *n* = 4,265 answered the open-ended questions.

^5^Widely used measures of emotion regulation and coping utilize a two-item assessment approach, and demonstrate construct and predictive validity (e.g., Carver, 1997; Garnefski and Kraaij, 2006; see also Eddy et al., 2019).

^6^We tested the convergent validity of our new ER and ES scales with two existing ER and ES scales in an independent educator sample (*N* = 340). We used structural equation modeling with maximum likelihood estimation and robust standard errors in Mplus 8.5. Both measures correlated highly with validated, long-form scales designed to tap facets of ER and ES. Our two-item perceived leader ER measure correlated with perceived leader ER assessed by: the 360° Trait Emotional Intelligence Questionnaire (TEIQue; Petrides, 2009) emotion regulation subscale (*r* = 0.88, *p <* 0.001), and the Self-Reported Emotional Intelligence Scale (SREIS; Brackett et al., 2006) emotion regulation subscale adapted for second-person ratings (*r* = 0.96, *p <* 0.001). Our two-item perceived leader ES measure correlated with perceived leader ES assessed by: the 360° TEIQue emotion management of others subscale (*r* = 0.42, *p <* 0.001), and the SREIS managing others’ emotions subscale adapted for second-person ratings (*r* = 0.97, *p <* 0.001).

^7^The Spearman-Brown method is recommended for testing the reliability of two-item scales (Eisinga et al., 2013).

^8^These outcomes were: emotional exhaustion, personal accomplishment, job satisfaction, positive and negative affect, and turnover intentions.

^9^We report the results of these additional CFAs in the Supplementary material for reference.

^10^The total sample was *N*_T1_ *=* 2,749 and *N*_T2_ = 1931. Like Study 1, we removed school leaders (*n*_T1_ = 94; *n*_T2_ = 58).

^11^For comparability with Study 1, we invited all school faculty and staff to participate in this study. Also, during the COVID-19 pandemic, we felt an ethical imperative to be inclusive in studying the well-being of all school adults.

^13^We also included other non-COVID-19 factors that might contribute to stress in this measure, such as finances and politics, but they are not included here, as these items are beyond the scope of the study.

^14^We also planned to include years working in education as a covariate. However, age and years working in education were highly correlated (*r* = 0.75, *p* < 0.001). So, we included age, but excluded years working in education, as age is among the most robust demographic correlates of occupational and personal well-being (Tay et al., 2014).

^15^The sample sizes for the severe vs. mild COVID-19 impact groups were *n* = 796 vs. *n* = 1,073, respectively.
