# Supplementary material for: Emotionally intelligent school leadership predicts educator well-being before and during a crisis
Source: Front Psychol. 2024 Feb 15;14:1159382. doi: 10.3389/fpsyg.2023.1159382 (PMC10903540; doi:10.3389/fpsyg.2023.1159382)
Supplement: Supplementary file 1 [file Data_Sheet_1.docx]

**Supplementary Materials**

**Study 1a: Results from a One-Factor CFA of Perceived School Leader Emotion Skills**

In our non-nested sample (*n* = 1793), a one-factor CFA of perceived leader emotion skills (ER and ES) showed poor model fit, *X*^2^ (2) = 967.21, *p* < .001; RMSEA = .53; CFI = .72; SRMR = .05. The standardized factor loadings ranged from .84 to .95. A one-factor model was not confirmed, suggesting that ER and ES should not be modeled as a single factor score.

**Study 1b: Results from a Two-Level One-Factor CFA of Perceived Leader Emotion Skills**

The two-level, one-factor model of perceived leader emotion skills showed inadequate fit, *X*^2^(4) = 1163.04, *p* < .001; RMSEA = .36; CFI = .83; SRMR_within_ = .05, SRMR_between_ = .08. At the educator level, the standardized factor loadings ranged from .86 to .92. At the school level, the standardized factor loadings ranged from .84 to 1.00. A one-factor model was rejected in favor of a better-fitting, theoretically-supported two-factor model of leader ER and ES.

**Study 1b: Results from a One-Level Two-Factor CFA of Perceived Leader Emotion Skills**

The single-level two-factor model showed good fit, *X*^2^(1) = 0.15, *p* = .70; RMSEA < .001; CFI = 1.00; SRMR < .001. The standardized factor loadings ranged from .95 to .96 for leader ER, and .95 to .96 for leader ES. The latent ER and ES scores correlated highly (*r* = .86, *p* < .001). A two-factor model of perceived leader emotion skills was supported by the data. We tested this model, so we could include the saved factor scores in our multilevel predictive analyses. We decided to parse the L1 and L2 variance in the predictive rather than measurement model. Note we were unable to conduct an SEM running the measurement and predictive models simultaneously with our Study 1b data, as we had insufficient model parameters for ML-SEMs. This is also why we ran single-level CFAs with the Study 1b data for emotional exhaustion and personal accomplishment, job satisfaction, positive and negaitve affect, and turnover intentions.

**Study 1b: Results from the Emotional Exhaustion and Personal Accomplishment CFA**

We conducted a single-level two-factor CFA for emotional exhaustion (EE) and personal accomplishment (PAcc) as they were measured with the same scale (MBI-ES) and they are theoretically related as components of burnout (Maslach et al., 2001). The CFA showed good model fit, *X*^2^(89) = 613.12, *p* < .001; RMSEA = .05; CFI = .95; SRMR = .05. The standardized factor loadings ranged from .58 to .86 for EE, and .36 to .70 for PAcc. Latent EE and PAcc scores were correlated (*r* = -.20, *p <* .001). A two-factor model was supported.

**Study 1b: Results from the Job Satisfaction CFA**

The single-level one-factor CFA for job satisfaction was just-identified (i.e., yielded zero degrees of freedom), and so the model fit statistics were not meaningful (e.g., the chi-square test of model fit = 0.00). However, the factor loadings are still valid (Muthén & Muthén, 2017). The standardized factor loadings ranged from .81 to .95. We also conducted a tau-equivalent model that generates model fit statistics for three-item measures by assuming the factor loadings are equal (Czerwiński & Atroszko, 2021). The tau-equivalent model converged, *X*^2^(4) = 95.73, *p* < .001; RMSEA = .10; CFI = .95; SRMR = .17. The SRMR was high, but the CFI and RMSEA fit was good and acceptable, respectively. A one-factor model of job satisfaction was retained.

**Study 1b: Results from the Positive and Negative Affect CFA**

We conducted a single-level two-factor CFA for positive affect (PA) and negative affect (NA) as both variables were measured with the same scale (PANAS-SF; Kercher, 1992; Mackinnon et al., 1999) and they are theoretically related. The CFA for PA and NA showed acceptable model fit, *X*^2^(34) = 857.01, *p* < .001; RMSEA = .10; CFI = 0.90; SRMR = .07. The standardized factor loadings ranged from .44 to .76 for PA, and .56 to .89 for NA. The latent PA and NA scores correlated moderately (*r* = -.37, *p <* .001). A two-factor model was supported.

**Study 1b: Results from the Turnover Intentions CFA**

The single-level one-factor CFA of turnover intentions was under-identified (i.e., yielded negative degrees of freedom), as it is a two-item measure, and so the model fit statistics were not interpretable. However, the factor loadings are valid. The standardized factor loadings were .77 and .73. For our purposes of entering a latent factor score of turnover intentions into a multilevel predictive analysis, we retained the one-factor model. This approach accounts for item-level measurement error, whereas a mean score of the items does not (MacCallum & Austin, 2000).

**Study 2: Results from a One-Factor CFA of Perceived School Leader Emotion Skills**

The one-factor CFA showed adequate model fit, though the RMSEA was high, *X*^2^(2) = 96.95, *p* < .001; RMSEA = .16; CFI = .97; SRMR = .03. The standardized factor loadings ranged from .76 to .80. A one-factor model was partially supported in Study 2, suggesting it may be possible to model ER and ES as a single factor score. Yet, the results from the two-factor model showed a superior fit to the data than the one-factor model, and as noted in Study 1, the two-factor model is better aligned with theory (Mayer et al., 2016), so the former model was retained.

References

Czerwiński, S. K., & Atroszko, P. A. (2021). A solution for factorial validity testing of three-item scales: An example of tau-equivalent strict measurement invariance of three-item loneliness scale. *Current Psychology*, *42*(2), 1652-1664. <https://doi.org/10.1007/s12144-021-01554-5>

Kercher, K. (1992). Assessing subjective well-being in the old-old: The PANAS as a measure of orthogonal dimensions of positive and negative affect. *Research on Aging*, *14*(2), 131-168. https://doi.org/10.1177/0164027592142001

MacCallum, R. C., & Austin, J. T. (2000). Applications of structural equation modeling in psychological research. *Annual review of psychology*, *51*(1), 201-226. <https://doi.org/10.1146/annurev.psych.51.1.201>

Mackinnon, A., Jorm, A. F., Christensen, H., Korten, A. E., Jacomb, P. A., & Rodgers, B. (1999). A short form of the Positive and Negative Affect Schedule: Evaluation of factorial validity and invariance across demographic variables in a community sample. *Personality and Individual differences*, *27*(3), 405-416. <https://doi.org/10.1016/S0191-8869(98)00251-7>

Maslach, C., Schaufeli, W. B., & Leiter, M. P. (2001). Job burnout. *Annual review of Psychology*, *52*(1), 397-422. <https://doi.org/10.1146/annurev.psych.52.1.397>

Muthén, L. K., & Muthén, B. O. (2017). *Mplus user’s guide* (8th ed.). Los Angeles, CA: Muthén & Muthén. <https://www.statmodel.com/download/usersguide/MplusUserGuideVer_8.pdf>
